# Targeting GLP-1R and IL-17A suppresses obesity-induced leukemia in an oncogenic *PTPN11* mutation–driven model

**DOI:** 10.1172/JCI202856

**Published:** 2026-06-23

**Authors:** Reuben Kapur, Linke Li, Rahul Kanumuri, Kanaka Sai Ram Padam, Baskar Ramdas, Chiranjeevi Pasala, Gabriela Chiosis, Lakshmi Reddy Palam, Ramesh Kumar, Satoshi Koyama, Pradeep Natarajan, Laura S. Haneline, Zhi Yu, Santhosh Kumar Pasupuleti

**Affiliations:** 1 Herman B Wells Center for Pediatric Research, Department of Pediatrics, Indiana University School of Medicine (IUSM), Indianapolis, Indiana, USA.; 2 Clinical and Translational Epidemiology Unit, Massachusetts General Hospital, Boston, Massachusetts, USA.; 3 Program in Medical and Population Genetics and the Cardiovascular Disease Initiative, Broad Institute of Harvard and MIT, Cambridge, Massachusetts, USA.; 4 Chemical Biology Program, Memorial Sloan Kettering Cancer Center, New York, New York, USA.; 5 Cardiovascular Research Center, Massachusetts General Hospital, Boston, Massachusetts, USA.; 6 Department of Medicine, Harvard Medical School, Boston, Massachusetts, USA.

**Keywords:** Hematology, Inflammation, Oncology, Hematopoietic stem cells, Leukemias, Obesity

## Abstract

Obesity is increasingly implicated in hematopoietic malignancies, yet its role in mutation-driven myeloid leukemias remains unclear. Using UK Biobank data from over 440,000 individuals, we found obesity traits including elevated BMI and waist-to-hip ratio were associated with type 2 diabetes, increased plasma IL-17A levels, reduced glucagon-like peptide 1 receptor (GLP-1R) expression, and heightened risk of myeloid malignancies. Transplantation of protein tyrosine phosphatase nonreceptor type 11 (*PTPN11*) (*Shp2^E76K/+^*) mutant hematopoietic stem/progenitor cells into obese mice demonstrated that metabolic inflammation accelerated leukemogenesis via myeloid cell expansion, lipid metabolic rewiring, IL-17A activation, and accumulation of M2-like tumor-associated macrophages (TAMs), accompanied by T cell exhaustion and impaired antigen presentation. Notably, dual therapy with an anti–IL-17A antibody and a GLP-1R agonist reversed these effects by reducing M2-like TAMs, restoring Ciita-dependent antigen presentation and Tyk2-mediated IFN-γ signaling, reactivating T cell responses, and reducing leukemic burden. These findings establish IL-17A–driven, metabolism-coupled immunosuppression as a mechanistic link between obesity and protein tyrosine phosphatase 2–mutant (SHP2-mutant) myeloid leukemias, highlighting a tractable therapeutic strategy for patients with obesity at high risk for other diseases and their complications.

## Abstract 2

Obesity fuels inflammation that accelerates PTPN11-mutant leukemia by suppressing anti-tumor immunity; targeting IL-17A and GLP-1R reverses this effect and reduces leukemic burden.

## Graphical abstract

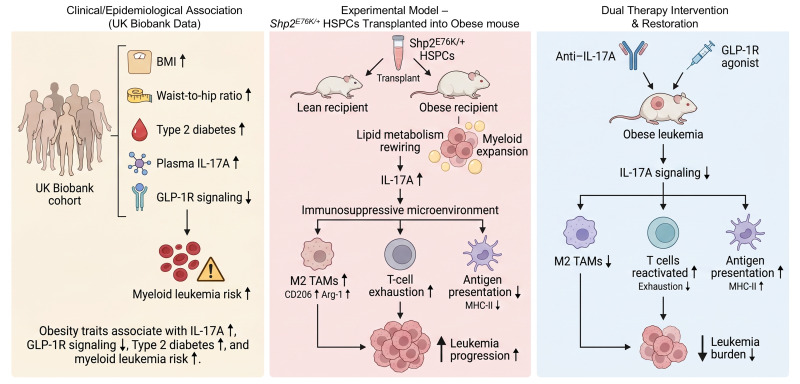

## Introduction

Obesity is a major global health challenge and a well-established risk factor for metabolic syndrome, type 2 diabetes mellitus (T2DM), and multiple cancers (1, 2). Increasing evidence implicates obesity in the development and progression of hematologic malignancies, particularly myeloid malignancies. Elevated BMI and waist-to-hip ratio (WHR) are associated with an increased risk of acute myeloid leukemia (AML), chronic myeloid leukemia (CML), poor survival, higher relapse rates, and treatment-related complications (3–6). In acute promyelocytic leukemia (APL), a subset of AML, obesity increases thrombohemorrhagic events, differentiation syndrome, and early death (6). Clinical studies, including the CALGB 10403 trial, have shown that individuals with leukemia and obesity faced higher mortality than those with normal BMI (<30 kg/m^2^) (7), and overweight individuals undergoing allogeneic hematopoietic stem cell transplantation (HSCT) exhibit reduced survival compared with nonoverweight patients (8, 9). Collectively, these observations highlight the profound effect of obesity on leukemia risk, treatment response, and prognosis, emphasizing the need to elucidate underlying biological mechanisms.

Although the precise mechanisms by which obesity contributes to leukemia initiation, progression, and relapse remain incompletely understood, emerging evidence suggests that cancer stem cells in individuals with obesity may reprogram adipocytes, which are highly accumulated in the bone marrow (BM) so-called fatty bone marrow (FBM) into cancer-associated adipocytes. These adipocytes secrete a variety of growth, inflammatory, fibrotic, and angiogenic factors, including TNF-α, adiponectin, IL-6, IL-1β, and VEGF, which can promote tumor growth and alter anticancer responses to chemotherapy (6, 10, 11).

The *PTPN11* gene encodes SH2-containing protein tyrosine phosphatase 2 (SHP2), a key regulator of hematopoietic differentiation, proliferation, and apoptosis (10–12). Germline *PTPN11* mutations occur in approximately 50% of patients with Noonan syndrome (NS), whereas somatic mutations are detected in approximately 37%–40% of juvenile myelomonocytic leukemia (JMML), approximately 5%–10% of acute myeloid leukemia (AML), and approximately 2%–5% of acute lymphoblastic leukemia (ALL) cases (11, 13, 14). These activating mutations constitutively stimulate Ras/MAPK signaling and promote myeloid transformation, and SHP2’s integration of cytokine, growth factor, and metabolic signals suggests that *PTPN11*-mutant hematopoietic cells may be particularly sensitive to systemic metabolic stressors (10, 12, 14). Despite these links, how obesity influences *PTPN11*-driven leukemogenesis remains poorly defined. Using integrated human genetic analyses and mouse models, we show that obesity-related traits such as elevated BMI and WHR are associated with increased T2DM, IL-17A signaling, reduced glucagon-like peptide 1 receptor (GLP-1R) expression, and a higher risk of myeloid leukemia. Transplantation of *Shp2^E76K/+^*-mutant mouse BM hematopoietic stem cell and progenitors (HSC/Ps) into obese mouse models demonstrated that systemic metabolic inflammation accelerated leukemogenesis via Th17/IL-17A signaling and M2-like tumor-associated macrophage (TAMs) accumulation and broad metabolic reprogramming. In particular, genes associated with lipid metabolism, cholesterol homeostasis, adipocytokine signaling, and atherosclerosis were upregulated, underscoring the convergence of metabolic dysfunction and myeloid-driven inflammation. Finally, we show that therapeutic targeting of anti–IL-17A antibody and a GLP-1R agonist reduced leukemic burden and partially restored normal hematopoiesis in obese mice bearing *Shp2^E76K/+^* cells. Together, these findings revealed a mechanistic link between metabolic dysregulation and *PTPN11*-driven leukemogenesis and highlight dual IL-17A blockade and GLP-1R agonism as promising therapeutic strategies for high-risk obese patients with *PTPN11* mutations.

## Results

### Associations of obesity with type 2 diabetes and myeloid leukemia incidence.

Obesity and metabolic dysfunction are increasingly recognized as critical modulators of hematopoietic malignancies, influencing both disease initiation and progression. However, the mechanisms by which obesity contributes to leukemia, particularly in the context of oncogenic mutations, remain poorly understood. Gain-of-function mutations in *PTPN11* (encoding SHP2) are implicated in pediatric leukemias such as JMML and AML, yet it is unclear whether metabolic traits like obesity or central adiposity modulate disease risk or progression in individuals harboring these mutations.

To address this, we leveraged large-scale human population data from the UK Biobank, comprising 440,982 unrelated participants with exome sequencing and anthropometric measurements for BMI or WHR (Table 1). The mean (SD) age of the participants was 56.6 (8.1) years, 45.9% were male, 54.1% were female, and 45.0% reported being ever-smokers. The mean BMI was 27.4 (4.8) kg/m^2^, and the mean WHR was 0.87 (0.1). There were 13,717 (3.1%) participants with baseline Metformin use, and among the 26,701 participants with prevalent diabetes (types 1 and 2) there were 13,282 (50%) participants using Metformin (Table 1). Using these data, we examined the association of BMI and WHR with incident type 2 diabetes (T2D) and myeloid malignancies. Over a median follow-up of 13.5 years (IQR: 12.63–14.29), the incidence of T2D was 6.6%. For myeloid malignancies, the incidence was 0.21% over a median follow-up of 13.6 years (IQR: 12.89–14.35). Both BMI (*P* < 0.001) and WHR (*P* < 0.001) were significantly associated with incident T2D after adjustment for age at enrollment, sex, ever-smoker status, and the first 10 principal components of genetic ancestry (Table 2). Each 1-SD increase in BMI was associated with a 1.95-fold increased risk of T2D (HR: 1.95, 95% CI: 1.93–1.96). Similarly, a 1-SD increase in WHR corresponded to a 1.46-fold increase in T2D (HR: 1.46, 95% CI: 1.46–1.47). We next examined the association of BMI and WHR with the incidence of myeloid malignancies (Table 3). A 1-SD increase in both BMI (HR: 1.09, 95% CI: 1.02–1.17; *P* = 0.02) and WHR (HR: 1.12, 95% CI: 1.03–1.23; *P* = 0.01) was significantly associated with an elevated risk of myeloid malignancies. Furthermore, individuals with obesity who also had T2D demonstrated a stronger risk (HR: 1.51, 95% CI: 1.08–2.13; *P* = 0.02) of myeloid malignancies (Table 3) compared with lean participants without diabetes. To account for the potential protective effect of Metformin on myeloid leukemia, we then conducted sensitivity analyses, adjusting for baseline Metformin use. After the adjustment, the positive association between a 1-SD increase in BMI (HR: 1.08, 95% CI: 1.00–1.16; *P* = 0.04) and WHR (HR: 1.11, 95% CI: 1.01–1.21; *P* = 0.03) with the incidence of myeloid leukemia remained robust (Table 3).

### PTPN11 mutations are associated with adiposity and obesity-related cancer risk.

**Figure 1:**
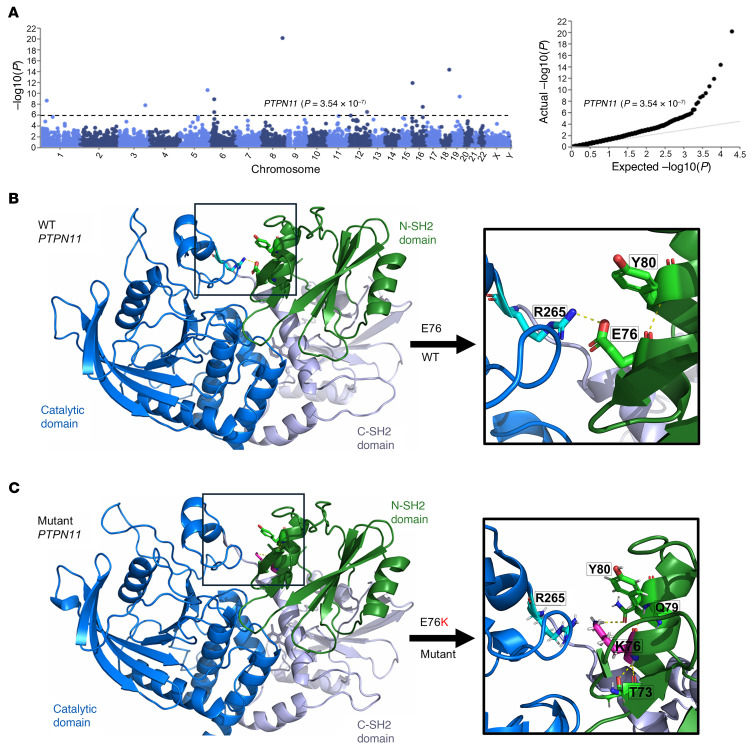
Association of *PTPN11* mutations with obesity-related traits and cancer prevalence. (**A**) Manhattan plot (left) and QQ plot (right) depicting gene-based association results for *PTPN11* rare variants with weight-related traits in the UK Biobank exome sequencing dataset. The Manhattan plot shows the –log_10_(*P*) values across chromosomes, highlighting *PTPN11* with a significant association *PTPN11* (*P* = 3.54 × 10^–7^). The QQ plot demonstrates deviation from the expected null distribution, confirming the significance association of *PTPN11* mutation with overweight. (**B** and **C**) Structural consequences of the SHP2 E76K mutation. Ribbon diagrams of WT (**B**) and E76K-mutant (**C**) SHP2 structures showing the N-SH2 (green), C-SH2 (violet), and catalytic PTP (blue) domains. In the WT, E76 forms a stabilizing hydrogen bond with R265, maintaining the protein in a closed, autoinhibited conformation. In the mutant, substitution of E76 with lysine abolishes the E76-R265 interaction and introduces new hydrogen bonds with T73, Q79, and Y80, favoring an open conformation of SHP2. This conformational shift exposes the catalytic domain, providing a structural basis for constitutive activation and highlighting the catalytic pocket as a potential therapeutic target.

Given the emerging connection between metabolic dysfunction and leukemogenesis and prior evidence linking *PTPN11* mutations to pediatric leukemias and poor outcomes in adult cancers, we hypothesized that *PTPN11* variants may contribute to adiposity and that obesity could amplify their oncogenic potential. To test this, we leveraged gene-based association results from 394,841 UK Biobank exomes in the Genebass database (15) using SAIGE-GENE (16). Rare missense variant burden in *PTPN11* was negatively associated with fat-free mass across all measured body compartments including both arms, both legs, trunk, and whole body (β ranges from –0.003 to –0.0025 SD, *P* < 5 × 10^–6^; Table 4). These associations between fat-free mass and rare missense variant burden of more common mutations in adults such as *TP53*, *FLT3*, and *NPM1* were not observed (Table 4). Consistent with a shift in body composition, our gene-based burden analysis also revealed an exome-wide significant association (*P* = 3.54 × 10^–7^) between *PTPN11* and body weight (Figure 1A). Burden analyses stratified by variant type further revealed that this signal was primarily driven by a missense variant set (*P* = 3.29 × 10^–7^), which includes variants such as p.Glu76Asp, p.Glu76Lys, p.Val432Met, p.Pro565Leu, p.Met508Val, and p.Asn308Asp (Supplemental Table 1 and Supplemental Data File 1), and it also showed a strong association with BMI (*P* = 1.93 × 10^–3^) (Supplemental Table 1; supplemental material available online with this article; https://doi.org/10.1172/JCI202856DS1). These findings highlight the functional importance of missense variants in modulating metabolic traits and implicate *PTPN11* mutations in the pathogenesis of obesity. To assess pathogenicity, mutant PTPN11 protein sequences were evaluated using sorting intolerant from tolerant (SIFT) and PolyPhen-2. Among these, the p.Glu76Asp and p.Glu76Lys variants scored as deleterious (tolerance index <0.01), whereas all tested variants were predicted to be pathogenic by PolyPhen-2 (Supplemental Table 2). Notably, we focused on the recurrent p.Glu76Lys (E76K) mutation, as it is among the most frequent and well-characterized *PTPN11* driver mutations in leukemia. Structural analysis further revealed that the WT SHP2 protein adopts a closed, autoinhibited conformation stabilized by a hydrogen bond between E76 in the N-SH2 domain and R265 in the catalytic domain (Figure 1B). In contrast, the E76K-mutant SHP2 structure displayed an open conformation with complete loss of the E76-R265 interaction, consistent with the experimentally solved crystal structure (Protein Data Bank [PDB]: 6CMS) (Figure 1C). Instead, the substituted Lys76 established new hydrogen bonds with T73, Q79, and Y80 within the N-SH2 domain, thereby destabilizing the closed interface. These structural rearrangements provide a mechanistic basis for how the E76K mutation disrupts autoinhibition and favors constitutive activation of SHP2. Collectively, these findings implicate *PTPN11* mutations in the genetic architecture of obesity and suggest that metabolic dysfunction may cooperate with oncogenic signaling in disease pathogenesis.

Apart from hematologic malignancies, *PTPN11* mutations are also prevalent in nonhematologic solid cancers and often portend a poor prognosis (17). To determine whether obesity may further modify this risk, we analyzed *PTPN11* mutations across solid cancer types in The Cancer Genome Atlas (TCGA) PanCancer cohort (*n* = 10,956). As summarized in Supplemental Figure 1A, the majority of cases involving *PTPN11* mutations occurred in uterine corpus endometrial carcinoma (UCEC), followed by colorectal adenocarcinoma (COAD), skin cutaneous melanoma (SKCM), and a spectrum of other malignancies at lower frequencies. This indicates that *PTPN11* mutations are broadly represented across diverse tumor types, with enrichment in specific cancer subtypes. Importantly, we found that *PTPN11* mutations were disproportionately represented among patients with a high BMI (>30 kg/m^2^). As shown in Supplemental Figure 1B, a greater number of patients harboring *PTPN11* variants had a markedly higher BMI than did *PTPN11* variant carriers who had a low BMI. Correspondingly, more than half (56.86%) of all individuals with *PTPN11* mutations were in the high-BMI subgroup (Supplemental Figure 1C). These findings suggest that *PTPN11* mutations associate with adiposity and may synergize with obesity to modulate cancer risk. Collectively, these data support a model in which rare *PTPN11* variants influence metabolic traits and show that co-occurrence with a high BMI may potentiate oncogenic signaling in diverse cancers, providing a potential mechanistic link between obesity, *PTPN11* mutation, and tumor susceptibility.

### Obesity induces the expansion and transformation of Ptpn11^E76K/+^-mutant HSC/Ps and exacerbates the development of an AML-like phenotype.

**Figure 2:**
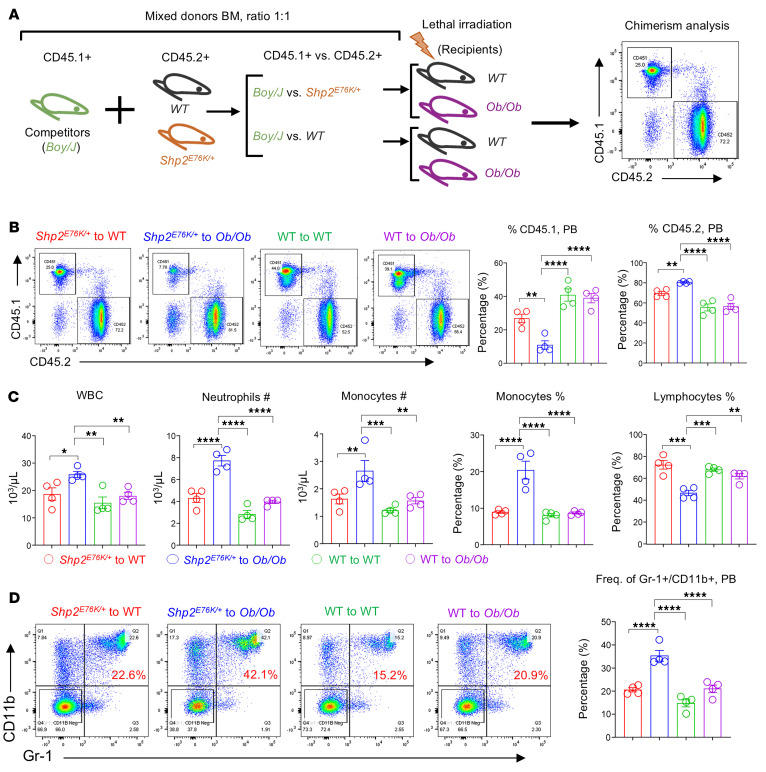
*Shp2^E76K/+^*-mutant cells outcompete the normal WT cells in *Ob/Ob* recipients. (**A**) Schematic of the competitive BMT assay. Donor BM cells from *Shp2^E76K/+^* or WT mice (CD45.2^+^) were mixed with Boy/J competitor (CD45.1^+^) BM cells (500,000:500,000) and then transplanted into lethally irradiated WT or *Ob/Ob* recipient mice. Donor-derived chimerism was observed using antibodies against CD45.1 or CD45.2. (**B**) Representative profiles of flow cytometry for donor chimerism in the PB of recipient mice and quantification of CD45.1^+^ cells and CD45.2^+^ in the PB of indicated recipient mice. (**C**) PB counts at 24 weeks after BMT from the indicated recipient mice. (**D**) Representative flow cytometric profile of myeloid cells (Gr-1^+^CD11b^+^) in the PB of the indicated recipient mice and frequency of Gr-1^+^CD11b^+^ myeloid cells in the PB of competitive transplant recipients over 24 weeks. *n* = 4 mice per group. Freq., frequency. **P < 0.05, **P < 0.005, ***P < 0.0005, ****P < 0.0001, by 1-way ANOVA followed by Tukey’s post hoc test. Data are presented as the mean ± SEM.

To investigate the mechanistic link between *PTPN11* mutations, obesity, and hematopoietic malignancy, we turned to mouse models with the *Shp2^E76K/+^* mutation, allowing us to examine how mutant HSC/Ps behave in lean versus obese BM environments. Using a competitive BM transplantation (BMT) assay that allows measurement of the expansion, survival, and engraftment of *Shp2^E76K/+^*-mutant cells, we transplanted a mixture of BM cells from WT mice or donor mice carrying the *Shp2^E76K/+^* mutation (CD45.2^+^) and BM cells from Boy/J competitor mice (CD45.1^+^) into lethally irradiated leptin-deficient *Lep^Ob/Ob^* (*Ob/Ob*) FBM mice or WT lean BM recipients (Figure 2A). Engraftment of *Shp2^E76K/+^*-mutant CD45.2^+^ cells and the frequencies of mature hematopoietic cells in the peripheral blood (PB) of recipient mice was monitored for 24 weeks following transplantation. *Ob/Ob* FBM mice transplanted with *Shp2^E76K/+^*-mutant BM cells showed increased engraftment of CD45.2^+^ cells and decreased engraftment of WT normal CD45.1^+^ cells in the PB compared with controls (Figure 2B). Measurement of PB counts exhibited that *Ob/Ob* FBM mice transplanted with *Shp2^E76K/+^*-mutant HSC/Ps had high absolute counts of WBCs and myeloid cells, including neutrophils and monocytes and decreased numbers of lymphocytes compared with other groups (Figure 2C). Similarly, increased frequencies of myeloid cells (Gr1^+^CD11b^+^) were observed in PB, BM, and spleens of *Ob/Ob* FBM mice transplanted with *Shp2^E76K/+^*-mutant HSC/Ps compared with control mice (Figure 2D and Supplemental Figure 2A). Additionally, *Ob/Ob* recipients of *Shp2^E76K/+^*-mutant HSC/Ps developed hepatosplenomegaly (Supplemental Figure 2, B and C). As expected, systemic metabolic abnormalities including increased body weight, hyperglycemia, and visceral adiposity were evident in all *Ob/Ob* mice regardless of whether they received WT or *Shp2^E76K/+^*-mutant HSC/Ps (Supplemental Figure 2, D–F), reflecting the underlying leptin deficiency. Importantly, while obesity established a permissive metabolic environment, leukemic progression characterized by splenomegaly, expansion of immature myeloid cells, and skewed hematopoietic differentiation was specifically driven by *Shp2^E76K/+^*-mutant HSC/Ps. Consistent with this, obese recipient mice transplanted with *Shp2^E76K/+^-*mutant cells showed significantly (median overall survival: 190 vs. 240 days, *P* = 0.03) reduced survival compared with lean controls (Supplemental Figure 2G).

**Figure 3:**
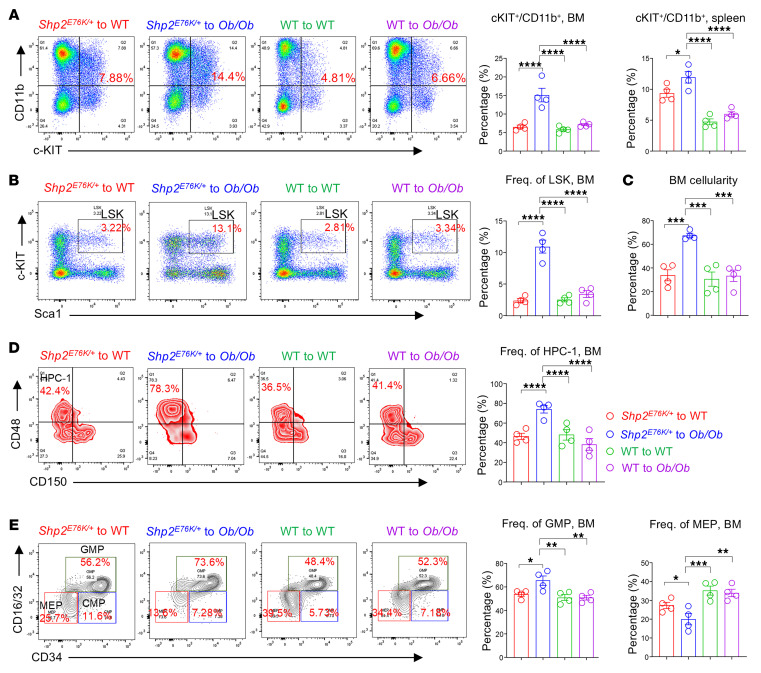
*Shp2^E76K/+^*-mutant myeloid blasts and HSC/Ps are expanded in *Ob/Ob* recipients. (**A**) Representative flow cytometric profiles of c-KIT^+^CD11b^+^ myeloid blasts from the indicated recipient mice and frequency of c-KIT^+^CD11b^+^ cells in BM and in spleens of competitive transplant recipients over 24 weeks. (**B**) Representative flow cytometric profile of LSK cells in BM from the indicated recipient mice and absolute number of LSK cells in BM and (**C**) BM cellularity of competitive transplant recipients over 24 weeks. (**D**) Representative flow cytometric profile of HPC1 cells (LSK CD48^+^CD150^–^) in BM from the indicated recipient mice and frequency of HPC1 cells in BM of competitive transplant recipients over 24 weeks. (**E**) Representative flow cytometric profile of HSC/Ps in BM and frequency of GMPs (Lin^–^c-KIT^+^CD16/32^+^CD34^+^) and MEPs (Lin^–^c-KIT^+^CD16/32^–^CD34^–^) in BM of competitive transplant recipients over 24 weeks. *n* = 4 mice per group. *P < 0.05, **P < 0.005, ***P < 0.0005, ****P < 0.0001, by 1-way ANOVA followed by Tukey’s post hoc test. Data are presented as the mean ± SEM.

Notably, higher frequencies of c-KIT^+^CD11b^+^ myeloid blasts (~14% of blasts) were observed in BM and spleens from *Ob/Ob* FBM mice transplanted with *Shp2^E76K/+^*-mutant HSC/Ps, consistent with the development of the early-onset AML-like phenotype compared with other groups (Figure 3A). Furthermore, the frequency of Lin^–^, Sca-1^+^, and c-KIT^+^ (LSK) cells, abnormal BM cellularity, frequency of hematopoietic progenitor cell 1cells (LSK CD48^+^CD150^–^) and granulocyte macrophage progenitors (GMPs) (c-KIT^+^CD16^+^CD32^+^CD34^+^ cells) were elevated in *Ob/Ob* FBM mice transplanted with *Shp2^E76K/+^*-mutant HSC/Ps compared with control groups (Figure 3, B–E). Notably, *Ob/Ob* FBM mice transplanted with *Shp2^E76K/+^*-mutant HSC/Ps also had a substantially higher abundance of inflammatory (F4/80^+^CD206^+^) TAMs compared with other groups (Supplemental Figure 3A). These findings suggest that in an obese FBM microenvironment, *Shp2^E76K/+^* HSC/Ps represent disease-initiating cells, which results in abnormal differentiation of HSC/Ps, self-renewal, and hyperproliferation of immature myeloid cells.

In obese FBM, HSC/Ps are frequently surrounded by mature immune cells, which can contribute to enhanced myelopoiesis and expansion of myeloid progenitors (18). Thus, the inflammatory obese BM microenvironment is likely to affect the growth and self-renewal of these cells in part by secreting inflammatory chemokines, lymphokines, and cytokines (19). To investigate whether obese FBM enhanced the systemic inflammation due to the presence of *Shp2^E76K/+^* mutation, serum cytokine levels were measured. Serum cytokine/chemokine analysis of *Ob/Ob* FBM mice transplanted with *Shp2^E76K/+^* BM cells showed elevated levels of proinflammatory cytokines, including IL-5, IL-6, and IL-17A and chemokines including CXCL-5 and CCL3 compared with control mice (Supplemental Figure 3B). To evaluate inflammatory signaling in the BM of patients with AML, we compared the expression of key cytokines and chemokines between control and AML samples. AML BM samples (Gene Expression Omnibus [GEO]: GSE1159) (20) exhibited higher expression of IL-17A, IL-6, IL-5, TNF, IL-1β, CCL3, and CCL20 relative to controls (Supplemental Figure 4). Consistent with these findings, prior studies have reported increased levels of IL-6, IL-17A, IL-1β, TNF, CCL2, and CCL3 in the BM plasma of patients with AML compared with healthy individuals (21–24). Together, these findings suggest that *Shp2^E76K/+^*-mutant HSC/Ps under proinflammatory conditions rapidly progressed to an AML-like phenotype and end up outcompeting the WT BM cells in *Ob/Ob* recipient mice compared with WT mice.

Leptin is a hormone secreted from fat cells such as adipose tissue, and it helps to regulate food intake and body weight, and plays a key role in proinflammatory immune responses, angiogenesis, and lipolysis (25). Both humans and mice with leptin (*Lep*) or the leptin receptor (*Lepr*) develop obesity, hypertension, dyslipidemia, T2DM, insulin resistance, cardiovascular disease (CVD), and some types of cancers (26). To assess how *Shp2^E76K/+^*-mutant HSC/Ps respond in a *Lepr-*deficient (*Db/Db*) environment, we performed competitive BMT of a mixture of BM cells from *Shp2^E76K/+^* donor mice (CD45.2^+^) and BM cells from Boy/J competitor mice (CD45.1^+^) into lethally irradiated *Db/Db* or WT recipient mice to determine whether *Shp2^E76K/+^* cells behave in a manner similar to those in *Ob/Ob* recipient mice or in a different manner (Supplemental Figure 5A). All the *Db/Db* recipient mice transplanted with *Shp2^E76K/+^*-mutant BM cells became moribund at 22 weeks after transplantation. Donor-derived CD45.2^+^ cell percentages derived from *Shp2^E76K/+^*-mutant mice were elevated in *Db/Db* recipient mice compared with percentages in WT recipients, similar to *Ob/Ob* recipient mice (Supplemental Figure 5B). Furthermore, Gr-1^+^CD11b^+^ myeloid cells derived from *Shp2^E76K/+^* (CD45.2^+^) donor cells were increased in the PB of *Db/Db* recipients compared with WT recipients (Supplemental Figure 5C). Hematologic analysis of PB counts showed that *Db/Db* mice transplanted with *Shp2^E76K/+^* BM had increased absolute counts and percentages of neutrophils and monocytes and a modest reduction in platelets compared with WT recipient mice (Supplemental Figure 5D). Hepatosplenomegaly and increased body weights were also noted in *Db/Db* mice transplanted with *Shp2^E76K/+^*-mutant cells (Supplemental Figure 5, E–G), a phenotype seen in *Ob/Ob* recipients as well. Notably, donor-derived CD45.2^+^ cells and Gr-1^+^CD11b^+^ mature myeloid cells in BM and spleens were elevated in the *Db/Db* recipient mice transplanted with *Shp2^E76K/+^*-mutant cells compared with WT mice (Supplemental Figure 5, H and I). Additionally, the frequencies of c-KIT^+^CD11b^+^ myeloid blasts derived from *Shp2^E76K/+^* (CD45.2^+^) donor cells were increased in the BM, spleen, and PB of *Db/Db* recipient mice compared with WT mice (Supplemental Figure 5J). We also noted that the frequency and the absolute number of LSK cells, BM cellularity, frequency, and absolute numbers of Lin-Sca-1^+^c-Kit^+^CD48^+^CD150^–^ (HPC1) cells and GMPs were increased and a profound decrease in megakaryocyte erythroid progenitors (MEPs) (c-KIT^+^CD16/32^–^CD34^–^ cells) was noted in *Db/Db* recipient mice transplanted with *Shp2^E76K/+^-*mutant cells compared with WT recipient mice (Supplemental Figure 6, A–D). Importantly, serum cytokine and chemokine analysis showed that, similar to *Shp2^E76K/+^* BM cells transplanted into *Ob/Ob* recipient mice, *Db/Db* recipient mice transplanted with same cells also showed elevated proinflammatory cytokines and chemokines such as IL-17A, granulocyte-CSF (G-CSF), and IL-12p70 (Supplemental Figure 6E). These results suggest that obesity-induced inflammation promoted the growth of *Shp2^E76K/+^*-mutant HSC/Ps, which rapidly developed into AML-like cells and showed signs of leukemogenesis in *Db/Db* recipient mice compared with WT mice.

### Obesity amplifies IL-17A–linked inflammatory, metabolic, and cellular reprogramming in Shp2^E76K/+^-driven BM.

**Figure 4:**
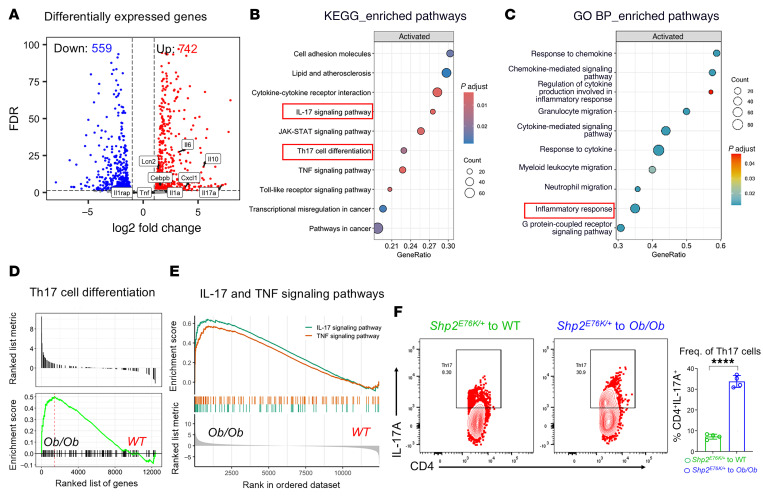
Obesity exacerbates transcriptomic dysregulation in *Shp2^E76K/+^*-mutant HSC/Ps. (**A**) Differential gene expression analysis comparing *Ob/Ob* mice bearing *Shp2^E76K/+^* HSC/Ps with WT mice bearing *Shp2^E76K/+^* HSC/Ps identified 559 significantly downregulated and 742 upregulated genes (*P*adj < 0.05, log_2_FC >1). (**B**) GSEA of KEGG pathways (Bonferroni-Hochberg–adjusted *P* < 0.05) and (**C**) GO biological processes (Bonferroni-Hochberg–corrected, *P*adj < 0.05) revealed key pathways altered in *Ob/Ob* mice bearing *Shp2^E76K/+^* HSC/Ps relative to WT counterparts. (**D** and **E**) Ranked GSEA plots showing enrichment of Th17 cell differentiation (**D**) and IL-17 and TNF signaling (**E**) pathways in *Ob/Ob* mice bearing *Shp2^E76K/+^* HSC/Ps compared with WT mice bearing *Shp2^E76K/+^* HSC/Ps. (**F**) Flow cytometric analysis of Th17 cells and frequency of Th17 (CD4^+^IL-17A^+^) cells in spleens of *Ob/Ob* mice bearing *Shp2^E76K/+^* HSC/Ps and WT mice bearing *Shp2^E76K/+^* HSC/Ps. *n* = 4 mice per group. ****P < 0.0001, by 2-tailed Student’s *t* test. Data are presented as the mean ± SEM.

**Figure 5:**
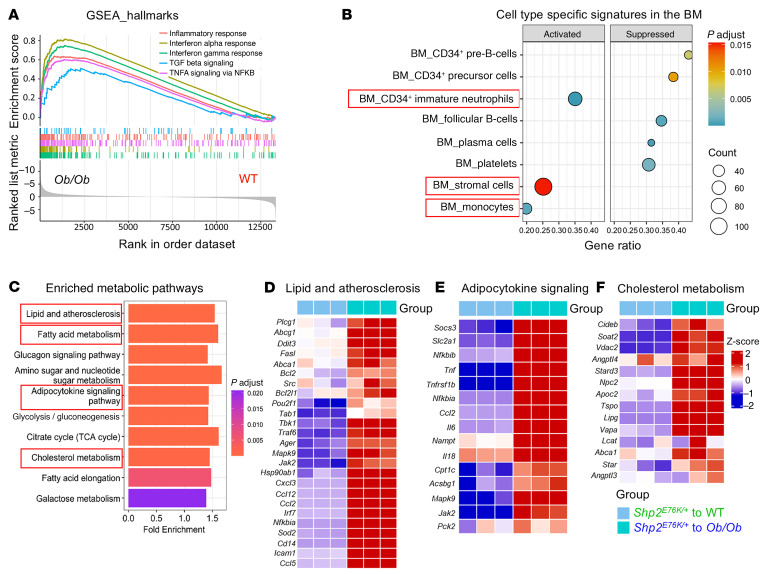
Transcriptomic pathway and gene expression changes in *Ob/Ob* mice bearing *Shp2^E76K/+^*-mutant HSC/Ps. (**A**) GSEA of RNA-seq data show significant activation of inflammatory and cancer hallmark pathways (NES >1, *P*adj < 0.05) in *Ob/Ob* mice bearing *Shp2^E76K/+^* HSC/Ps. (**B**) Altered BM cell-type–specific gene signatures identified by GSEA (Bonferroni-Hochberg correction, *P*adj < 0.05) in *Ob/Ob* mice bearing *Shp2^E76K/+^* HSC/Ps versus WT recipient mice. (**C**) Metabolic pathway enrichment analysis with heatmap visualization of gene expression changes involved in (**D**) lipid metabolism and atherosclerosis, (**E**) adipocytokine signaling, and (**F**) cholesterol metabolism in *Ob/Ob* mice bearing *Shp2^E76K/+^* HSC/Ps. Expression data are represented as *z* scores normalized across groups.

To dissect transcriptional alterations induced by obesity in the context of *PTPN11*-mutant hematopoiesis, we compared BM transcriptomes from WT and *Ob/Ob* mice transplanted with *Shp2^E76K/+^*-mutant HSC/Ps. Differential expression analysis identified 742 genes upregulated and 559 downregulated in *Ob/Ob* recipients compared with WT controls (FDR <0.05; Figure 4A). Strikingly, the most prominently upregulated genes included canonical inflammatory mediators such as *Il17a*, *Il17ra*, *Il1B*, *Ccl2*, *Ccl20*, *Tnf*, and *Il6*, pointing to a fatty marrow environment in which obesity and oncogenic Shp2 signaling converged to fuel a proinflammatory niche. Pathway enrichment analyses reinforced this conclusion. Kyoto Encyclopedia of Genes and Genomes (KEGG) analysis highlighted IL-17 signaling, Th17 cell differentiation, cytokine-cytokine receptor interactions, JAK/STAT signaling, and TNF signaling as the top activated pathways (Figure 4B). Likewise, Gene Ontology (GO) analysis revealed marked enrichment of genes linked to inflammatory responses, neutrophil migration, chemokine signaling, and GPCR-mediated signaling (Figure 4C). Gene set enrichment analysis (GSEA) independently validated robust enrichment of Th17, IL-17, and TNF signaling pathways in *Ob/Ob* recipients (Figure 4, D and E). In line with these transcriptional signatures, flow cytometric analysis revealed a marked expansion of Th17 cells (IL-17A^+^CD4^+^) in the *Ob/Ob* recipients compared with WT controls (Figure 4F). Additional hallmark pathways enriched in *Ob/Ob* recipients transplanted with *Shp2^E76K/+^*-mutant HSC/Ps included the inflammatory response, TGF-β signaling, and TNF signaling via NF-κB (Figure 5A). At the cellular level, obesity reinforced myeloid bias in the setting of *Ptpn11*-mutant hematopoiesis. As shown in Figure 2, B–D, and Figure 3, D and E, *Ob/Ob* recipients transplanted with *Shp2^E76K/+^* HSC/Ps displayed elevated neutrophils, monocytes, and HSC/Ps, accompanied by a reduction in lymphocytes and platelets. Cell-type–specific enrichment of bulk BM RNA-seq analysis further confirmed the expansion of inflammatory myeloid cell populations, with strong activation signatures in CD34^+^ immature neutrophils, monocytes, and stromal cells, together with suppression of B cell subsets and platelets (Figure 5B). In addition to immune remodeling, obesity triggered profound metabolic rewiring of leukemic BM. Pathway analysis identified enrichment of lipid and atherosclerosis signaling, fatty acid metabolism, adipocytokine signaling, glycolysis/gluconeogenesis, and cholesterol metabolism (Figure 5, C–F). These findings suggest that obesity integrated inflammatory signaling with altered metabolic pathways to further potentiate *Shp2^E76K/+^*-driven leukemogenesis. Together, these data demonstrate that obesity synergized with *Ptpn11*-mutant hematopoiesis to amplify IL-17A–linked inflammation, skew hematopoiesis toward proleukemic myeloid lineages, suppress adaptive immune cell subsets, and reprogram BM metabolism, thereby reinforcing a microenvironment conducive to leukemic progression.

Serum cytokine profiling of *Shp2^E76K/+^*-mutant HSC/Ps transplanted into *Ob/Ob*, and *Db/Db* recipient mice revealed markedly elevated the expression of IL-17A compared with WT recipients. To extend these findings to humans, we analyzed circulating IL-17A levels in plasma from 50,000 UK Biobank participants with available proteomics data who were free of hematological malignancies and myeloid leukemia at baseline. Among 2,498 participants (5.26%) with both diabetes and obesity defined by WHR, the mean plasma IL-17A levels were 0.14 SD higher (95% CI: 0.10–0.18, *P* < 0.001) compared with individuals without either condition or with only one (Table 5). Elevated IL-17A levels were also observed in participants with diabetes versus those without diabetes (0.12 SD higher, 95% CI: 0.08–0.16) and in individuals with obesity versus those without obesity (0.03 SD higher, 95% CI: 0.01–0.05). Similarly, participants with both obesity and diabetes had reduced average plasma GLP-1R levels compared with participants who were neither obese nor diabetic, nor both (ß ranges from –0.06 to –0.04, *P* < 0.05, Table 6).

IL-17A, a proinflammatory cytokine predominantly secreted by Th17 cells, signals through the IL-17RA–IL-17RC receptor complex and plays a dual role in host defense and pathology. While essential for protection against extracellular bacterial and fungal infections, dysregulated IL-17A promotes chronic inflammation, autoimmunity, and tumor progression (27–29). Notably, IL-17A has been shown to induce macrophage polarization toward an M2-like immunosuppressive phenotype (30). Consistently, elevated serum IL-17A in patients with various malignancies correlates with a poor prognosis (31). Clinically, 3 FDA-approved IL-17A–targeting antibodies — secukinumab, ixekizumab, and brodalumab — are used for autoimmune diseases, with secukinumab recently approved for pediatric patients aged 2 years or older for the treatment of juvenile psoriatic arthritis (32, 33).

Likewise, GLP-1R agonists have emerged as potent modulators of glucose homeostasis, body weight, and inflammation. Beinaglutide, a short-acting recombinant GLP-1R agonist that is structurally nearly identical to endogenous human GLP-1, provides rapid postprandial glucose control, robust weight loss, and antiinflammatory effects (34–37). Taken together, these observations led us to hypothesize that combined IL-17A inhibition and GLP-1R activation could synergistically suppress leukemic expansion, limit M2-like macrophage–mediated immunosuppression, and restore normal hematopoiesis in obese mice harboring *Shp2^E76K/+^*-mutant HSC/Ps.

### The combination of GLP-1R agonist and anti–IL-17A antibody therapy inhibits exacerbation of Shp2^E76K/+^-mutant HSC/Ps in Ob/Ob mice.

**Figure 6:**
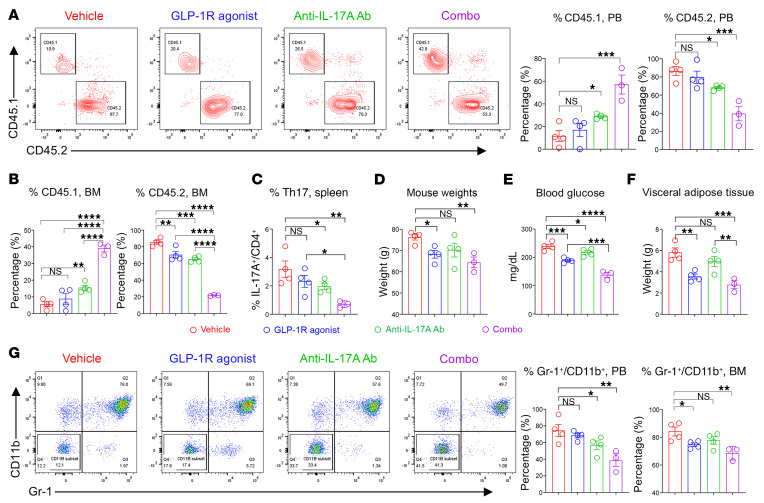
Combination therapy with GLP-1R agonist and an anti–IL-17A antibody reduces myeloid cell infiltration and improves metabolic parameters in *Ob/Ob* mice bearing *Shp2^E76K/+^*-mutant HSC/Ps. (**A**) Representative flow cytometry plots of donor chimerism (CD45.1^+^CD45.2^+^) in the PB of recipient mice after 30 days of the indicated drug treatment. Graphs in **A** show quantification of CD45.1^+^ cells and CD45.2^+^ cells in PB and (**B**) quantification of CD45.1^+^ cells and CD45.2^+^ cells in BM after 30 days of the indicated drug treatment. (**C**) Quantification of IL-17A^+^CD4^+^ cells in spleens after 30 days of the indicated drug treatment. Mouse (**D**) body weights, (**E**) fasting blood glucose levels, and (**F**) visceral adipose white tissue weights after 30 days of the indicated drug treatments. (**G**) Representative flow cytometric profile of myeloid cells (Gr-1^+^CD11b^+^) in the PB of recipient mice after 30 days of the indicated drug treatment and quantification of Gr-1^+^CD11b^+^ double-positive cells in PB and BM after 30 days of the indicated drug treatment. *n* = 3–4 mice per group. *P < 0.05, **P < 0.005, ***P < 0.0005, and ****P < 0.0001, by 1-way ANOVA followed by Tukey’s post hoc test. Data are presented as the mean ± SEM.

**Figure 7:**
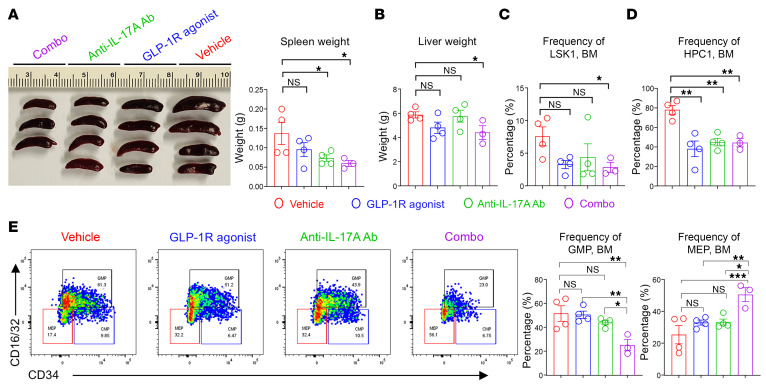
Combination treatment with a GLP-1R agonist and anti–IL-17A antibody attenuates splenomegaly and normalizes aberrant HSC/P populations in *Ob/Ob* mice bearing *Shp2^E76K/+^*-mutant HSC/Ps. (**A**) Spleen images and quantification of spleen weights, (**B**) liver weights, (**C**) LSK cells, and (**D**) HPC-1 cells. (**E**) Representative flow cytometry plots of HSC/Ps in BM of recipient mice after 30 days of the indicated drug treatment and quantification of GMPs and MEPs in BM after 30 days of the indicated drug treatment. *n* = 3–4 mice per group. *P < 0.05, **P < 0.005, and ***P < 0.0005, by 1-way ANOVA followed by Tukey’s post hoc test. Data are presented as the mean ± SEM.

To determine whether simultaneous targeting of metabolic and inflammatory pathways could mitigate obesity-driven exacerbation of *Shp2^E76K/+^*-mutant hematopoiesis, we performed competitive BMT again, as shown in Figure 2A, using a 1:1 mixture of *Shp2^E76K/+^* donor BM cells (CD45.2^+^) and competitor Boy/J BM cells (CD45.1^+^). These cells were transplanted into lethally irradiated *Ob/Ob* or WT recipients (Figure 2A). By week 8 after transplantation, the *Ob/Ob* recipients showed markedly enhanced engraftment of *Shp2^E76K/+^* donor HSC/Ps compared with WT controls. The mice were then randomized and treated for 30 days with beinaglutide, a GLP-1R agonist (3 mg/kg, s.c.), an anti–IL-17A antibody (200 μg/mouse, i.p., 3 times weekly), or the combination of both. Dosing regimens were selected on the basis of prior studies demonstrating efficacy with minimal toxicity (38, 39). Combination therapy markedly increased the proportion of normal CD45.1^+^ competitor cells and concomitantly reduced *Shp2^E76K/+^*-mutant CD45.2^+^ cells in both the PB and BM of *Ob/Ob* recipients (Figure 6, A and B). Furthermore, Th17 cell (IL-17A^+^CD4^+^) frequencies, body weight, fasting blood glucose levels, and visceral adipose tissue mass was reduced in the combination therapy group compared with the other groups (Figure 6, C–F). Single-agent treatments yielded partial reductions in mutant myeloid (Gr-1^+^CD11b^+^) populations, whereas combination therapy showed substantial suppression in both PB and BM (Figure 6G). Notably, combination therapy ameliorated hepatosplenomegaly, a hallmark of AML and JMML, in *Ob/Ob* recipients (Figure 7, A and B) and reduced leukemic stem/progenitor compartments in the BM, including LSK cells, HPC1 cells, and GMPs, while increasing MEP frequency (Figure 7, C–E). Together, these results demonstrate that dual targeting of IL-17A–driven inflammation and metabolic dysregulation not only limited the expansion of *Shp2^E76K/+^*-mutant HSC/Ps in obese hosts but also promoted the restoration of normal hematopoiesis, indicating the therapeutic potential of this combinatorial approach for obesity-associated leukemia pathogenesis.

### Combined GLP-1R agonism and IL-17A blockade most effectively reverses inflammatory and myeloid reprogramming in obese mice bearing Shp2^E76K/+^ cells.

**Figure 8:**
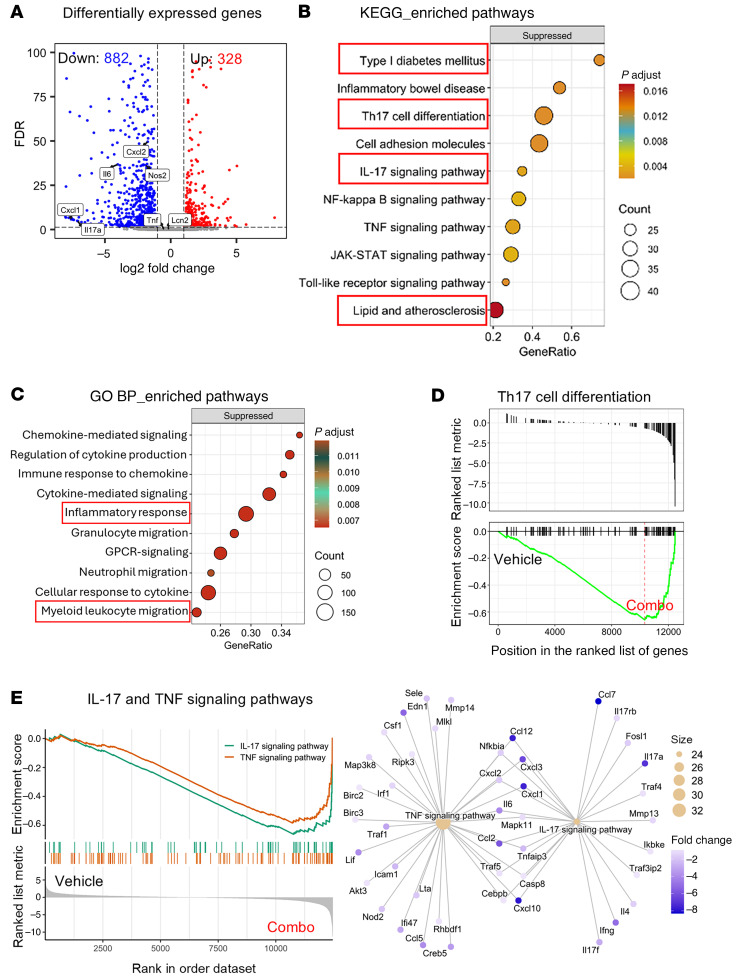
Combination therapy with a GLP-1R agonist and an anti–IL-17A antibody reverses inflammatory and myeloid dysregulation in *Ob/Ob* mice bearing *Shp2^E76K/+^* HSC/Ps. (**A**) Differential gene expression analysis comparing *Ob/Ob* mice bearing *Shp2^E76K/+^* HSC/Ps treated with the Combo therapy (GLP-1R agonist plus anti–IL-17A antibody) versus mice treated with vehicle revealed 882 significantly downregulated and 328 upregulated genes (*P*adj < 0.05). (**B** and **C**) GSEA of (**B**) KEGG pathways and (**C**) GO biological processes identified significantly enriched pathways in the Combo-treated group compared with the vehicle-treated group (Bonferroni-Hochberg *P*adj < 0.05). (**D** and **E**) Ranked GSEA plots show suppression of inflammatory pathways, including Th17 cell differentiation (**D**), IL-17 signaling, and TNF signaling (**E**) in Combo-treated mice relative to vehicle controls (NES >1, *P*adj < 0.05). Category-network plot on the right in **E** shows network visualization of differentially expressed genes (DEGs) associated with the IL-17 and TNF signaling pathways. Node size corresponds to gene connectivity, and node color represents relative expression changes.

**Figure 9:**
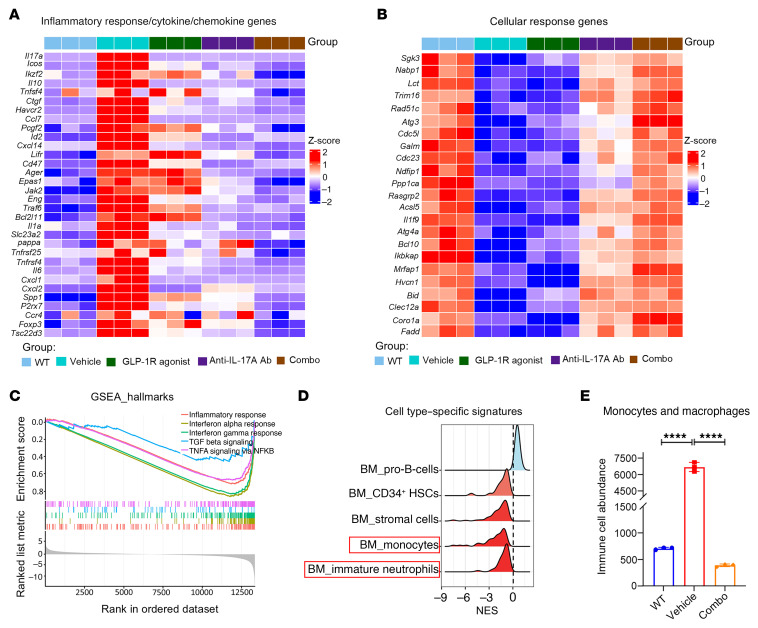
Combined GLP-1R agonism and IL-17A blockade restores BM immune homeostasis in *Ob/Ob* mice harboring *Shp2^E76K/+^* HSC/Ps. (**A** and **B**) Heatmap of normalized *z* scores depicting the expression levels of key inflammatory response genes, cytokines, and chemokines (**A**) and related cellular response profiles (**B**) across treatment groups. (**C**) GSEA of inflammatory and cancer hallmark pathways and (**D**) enrichment of BM cell-type–specific gene signatures highlight the molecular effect of combination therapy compared with vehicle-treated *Ob/Ob* mice bearing *Shp2^E76K/+^* HSC/Ps. (**E**) Quantification of relative monocyte and macrophage abundance in BM across treatment groups shows significant reductions in the combination group compared with vehicle. ****P < 0.0001, by 1-way ANOVA followed by Tukey’s post hoc test. Data are presented as the mean ± SEM.

To define the molecular effect of the GLP-1R agonist, the anti–IL-17A antibody, and their combination in obesity-driven, *Ptpn11*-mutant hematopoiesis, we compared BM transcriptomes from *Ob/Ob* mice bearing *Shp2^E76K/+^* HSC/Ps treated with vehicle, a GLP-1R agonist, an anti–IL-17A antibody, or both agents (Combo treatment). Whimle both monotherapies partially attenuated inflammatory gene expression, Combo treatment produced the most extensive transcriptional reprogramming, with 882 genes downregulated and 328 upregulated relative to the vehicle-treated HSC/Ps (log_2_ fold change [FC]>|1|, FDR < 0.05; Figure 8A). Pathway enrichment analysis demonstrated that Combo therapy most strongly suppressed immune and inflammatory signaling cascades. KEGG pathways were downregulated in the Combo group, including Th17 cell differentiation, IL-17 and TNF signaling, JAK/STAT signaling, NF-κB signaling, cytokine-cytokine receptor interaction, and lipid/atherosclerosis signaling (Figure 8B). GO enrichment analysis corroborated inhibition of the inflammatory response, cytokine-mediated signaling, granulocyte/neutrophil migration, and myeloid leukocyte chemotaxis (Figure 8C). Ranked GSEA confirmed a marked downregulation of Th17 cell differentiation (Figure 8D) and attenuation of IL-17/TNF signaling pathways (normalized enrichment score [NES] >1, adjusted *P* [*P*adj] < 0.05; Figure 8E), with network analysis revealing coordinated suppression of *Il17a*, *Il17re*, *Tnf*, *Cxcl1*, *Ccl2*, *Mmp13*, and *Socs3*. Heatmap profiling across all treatment arms showed that GLP-1R agonist or anti–IL-17A monotherapy each reduced subsets of inflammatory cytokine/chemokine genes (*Il1b*, *Ccl3*, *Ccl4*, *Cxcl2*, *Cxcl5*, *Tnf*) and cellular response genes, whereas Combo therapy broadly normalized the expression toward WT-like levels (Figure 9, A and B). GSEA of hallmark pathways mirrored this pattern, with the strongest suppression of inflammatory response, IFN-α/IFN-γ signaling, TGF-β signaling, and TNF signaling via NF-κB in the Combo group (Figure 9C). Cell-type–specific enrichment revealed that Combo therapy markedly decreased CD34^+^ immature neutrophils and monocytes, suppressed stromal cell activation, and partially restored the B cell compartment (Figure 9D). A reduction in BM monocytes/macrophage cell abundance was observed through deconvolution analysis in the Combo group compared with the vehicle-treated group (Figure 9E), which is consistent with our flow cytometric analysis (Figure 6G). In addition to immune reprogramming, Combo therapy profoundly corrected metabolic dysregulation in the BM. Vehicle-treated *Ob/Ob* mice transplanted with *Shp2^E76K/+^* HSC/Ps showed increased expression of genes in pathways related to lipid metabolism, fatty acid metabolism, adipocytokine signaling, atherosclerosis, and cholesterol metabolism (Figure 5, C–F). Combo therapy restored the expression of these metabolic genes toward baseline levels, normalizing fatty acid and cholesterol handling, suppressing adipocytokine-mediated proinflammatory signaling, and reversing the transcriptional programs associated with vascular and systemic metabolic dysfunction–related gene expression compared with other groups (Supplemental Figure 7, A and B). Collectively, these findings demonstrate that, while GLP-1R agonism or IL-17A blockade alone could partially counter obesity-driven IL-17/TNF–dominated inflammatory programs in *Shp2^E76K/+^* hematopoiesis, their combination most effectively dismantled these networks, normalized immune cell composition, and suppressed proatherogenic metabolic signatures, highlighting a potentially synergistic therapeutic avenue for obesity-associated leukemia progression.

### Combination therapy with GLP-1R agonist and IL-17A blockade reverses obesity and Shp2^E76K/+^-driven macrophage expansion, diminishes M2-like macrophages and T cell exhaustion, and restores cytotoxic T cell immunity.

**Figure 10:**
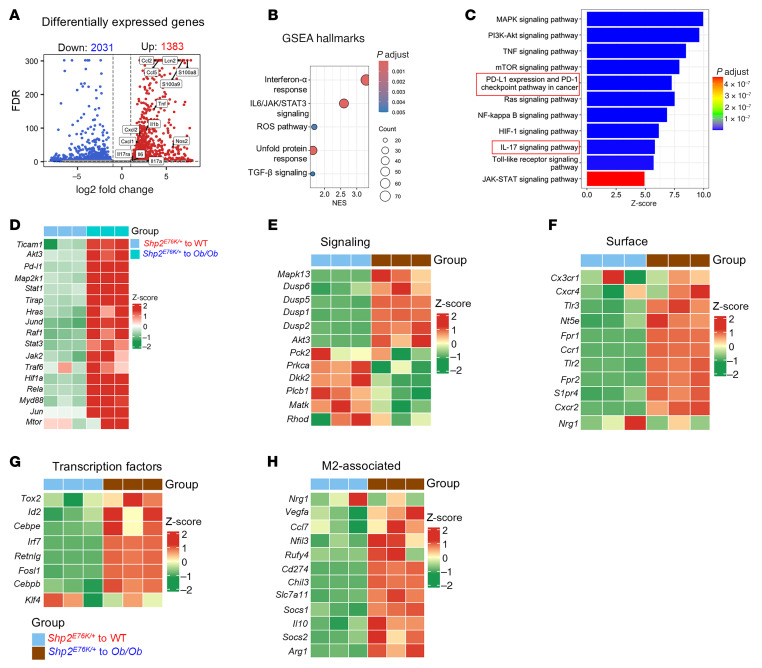
*Shp2^E76K/+^* HSC/Ps transplantation drives inflammatory and metabolic dysregulation in ATMs of *Ob/Ob* mice. (**A**) Differential gene expression analysis of sorted F4/80*^+^* ATMs from *Ob/Ob* mice transplanted with *Shp2^E76K/+^* versus WT HSC/Ps, showing 2,031 downregulated (Down) and 1,383 upregulated (Up) genes (*P*adj < 0.05). (**B**) GSEA enrichment analysis (Bonferroni-Hochberg–corrected *P*adj < 0.05) highlighting inflammatory and metabolic dysfunction signatures in *Ob/Ob* mice transplanted with *Shp2^E76K/+^* ATMs relative to WT. (**C** and **D**) Pathway analysis demonstrating enrichment of PD-1/PD-L1 checkpoint signaling and IL-17 pathways (**C**) with a corresponding heatmap of PD-1/PD-L1 pathway gene expression (**D**). (**E**–**G**) Heatmaps showing representative DEGs in ATMs, including signaling mediator, cell-surface, and transcription factor genes. (**H**) Heatmap displaying elevated expression of M2-associated macrophage markers in *Ob/Ob* mice transplanted with *Shp2^E76K/+^* HSC/Ps compared with WT.

Given the pronounced systemic and hematopoietic inflammatory signatures observed in *Ob/Ob* recipients of *Shp2^E76K/+^*-mutant HSC/Ps (Figures 6 and 7), we next examined adipose tissue macrophages (ATMs), a major driver of chronic inflammation in obesity-associated myeloproliferation. Consistent with enhanced visceral adiposity (Supplemental Figure 2F), these mice exhibited a marked expansion of F4/80^+^CD206^+^ ATMs (Supplemental Figure 3A). To define the molecular basis of this expansion, we performed RNA-seq on sorted F4/80^+^ ATMs. Transcriptomic profiling showed 2,031 downregulated and 1,383 upregulated genes in F4/80^+^ ATMs in *Ob/Ob* mice transplanted with *Shp2^E76K/+^*-mutant HSC/Ps compared with WT controls (Figure 10A). GSEA analysis identified enrichment of inflammatory and stress-related programs, including IFN-α/-γ responses, IL-6/JAK/STAT3 signaling, ROS production, the unfolded protein response, and TGF-β signaling in F4/80^+^ ATMs in *Ob/Ob* mice harboring *Shp2^E76K/+^*-mutant cells relative to WT controls (Figure 10B). Pathway analysis further revealed activation of the IL-17 and programmed cell death protein 1/programmed death–ligand 1 (PD-1/PD-L1) signaling cascades, with increased expression of *Pdl1*, *Myd88*, *Raf1*, *Rela*, and *Traf6* (Figure 10, C and D). GO analysis confirmed the upregulation of genes involving macrophage activation and functional programs, spanning signaling molecules (*Akt3*, *Mapk13*, *Dusp1*, *Dusp5*), surface receptor and chemokine molecules (*Cxcr4*, *Ccr1*, *Tlr2*, *Cxc3cr1*, *Cxcr4*, *Fpr1*, *Nt5e*, *Cxcr2*, *S1pr4*), and transcription factors (*Id2*, *Tox2*, *Fosl1*, *Cebpb*, *Irf7*) (Figure 10, E–G). Additionally, F4/80^+^ ATMs in *Ob/Ob* mice harboring *Shp2^E76K/+^*-mutant HSC/Ps led to increased expression of M2-like macrophage–associated genes, including *Arg1*, *Cd274* (encoding PD-L1), *Chil3*, *Il10*, *Socs1*, and *Nfil3* (Figure 10H). Collectively, these findings demonstrate that *Shp2^E76K/+^* HSC/Ps profoundly remodeled the ATM compartment, driving polarization toward an inflammatory and immunosuppressive state.

**Figure 11:**
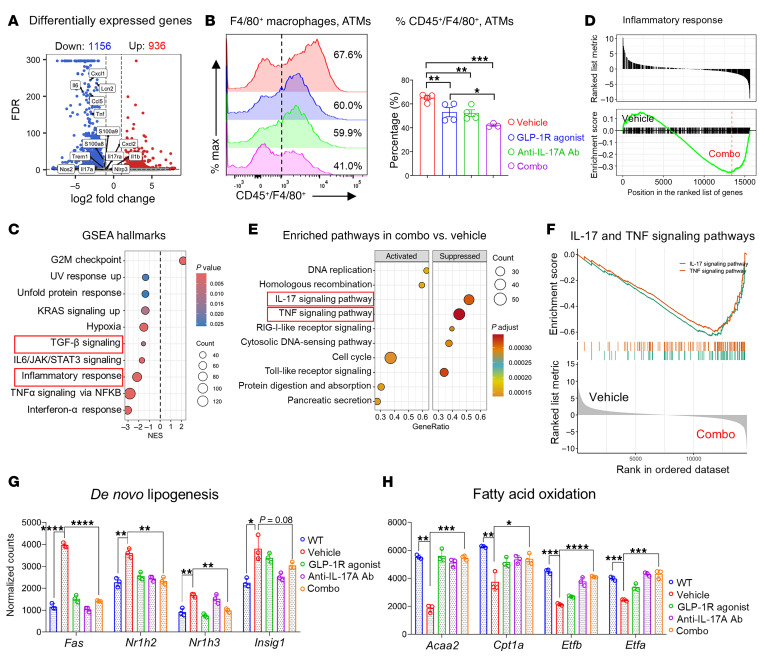
Combination of GLP-1R agonist and anti–IL-17A antibody therapy reverses inflammatory and metabolic dysregulation in ATMs of *Ob/Ob* mice bearing *Shp2^E76K/+^*-mutant HSC/Ps. (**A**) Differential gene expression analysis of ATMs from Combo-treated versus vehicle-treated *Ob/Ob* mice, identifying 1,156 downregulated and 936 upregulated genes. (**B**) Flow cytometry plots and quantification of CD45*^+^*F4/80*^+^* ATMs following vehicle or Combo therapy. (**C** and **D**) GSEA hallmark pathway enrichment showing reversal of inflammatory response and metabolic signatures in ATMs upon Combo therapy. (**E** and **F**) KEGG pathway analysis demonstrates suppression of IL-17, TNF, and DNA repair pathways, along with other metabolic and immune-related programs, in ATMs from Combo-treated mice. (**G** and **H**) Expression of de novo lipogenesis genes (*Fas*, *Nr1h2*, *Nr1h3*, and *Insig1*) and fatty acid oxidation genes (*Acaa2*, *Cpt1a*, *Etfb*, and *Etfa*) in ATMs from WT and *Ob/Ob* mice bearing *Shp2^E76K/+^* HSC/Ps and treated with the indicated drugs. *n* = 3–4 mice per group. *P < 0.05, **P < 0.005, ***P < 0.0005, and ****P < 0.0001, by 1-way ANOVA followed by Tukey’s post hoc test. Data are presented as the mean ± SEM.

We next tested whether dual GLP-1R agonism and IL-17A blockade could reverse these changes. Compared with vehicle treatment, Combo therapy reprogrammed the ATM transcriptome, with 1,156 genes downregulated and 936 upregulated (Figure 11A) and a substantial reduction in F4/80^+^ ATM abundance (Figure 11B). GSEA analysis revealed suppression of proliferative and inflammatory programs, including G2M checkpoint, UV response, KRAS signaling, hypoxia, TGF-β, IL-6/JAK/STAT3, and inflammatory response signaling pathways (Figure 11, C and D). KEGG enrichment analysis further demonstrated marked inhibition of IL-17 and TNF signaling, which are 2 central mediators of obesity-driven myeloproliferation, together with reductions in DNA replication and homologous recombination activity (Figure 11, E and F). Importantly, metabolic pathway analysis showed that ATMs from *Ob/Ob* mice exhibited upregulation of de novo lipogenesis and glycolysis-associated genes alongside reduced expression of fatty acid oxidation genes, consistent with a metabolically activated, inflammatory state. Although either GLP-1R agonism or IL-17A blockade alone produced modest effects, the Combo therapy robustly normalized ATM metabolism, suppressing lipogenic and glycolytic programs, while restoring fatty acid oxidation gene activity (Figure 11, G and H, and Supplemental Figure 7C). These findings show that dual targeting of metabolic and inflammatory signaling is required to fully reprogram macrophage function and overcome obesity-driven myeloproliferation.

**Figure 12:**
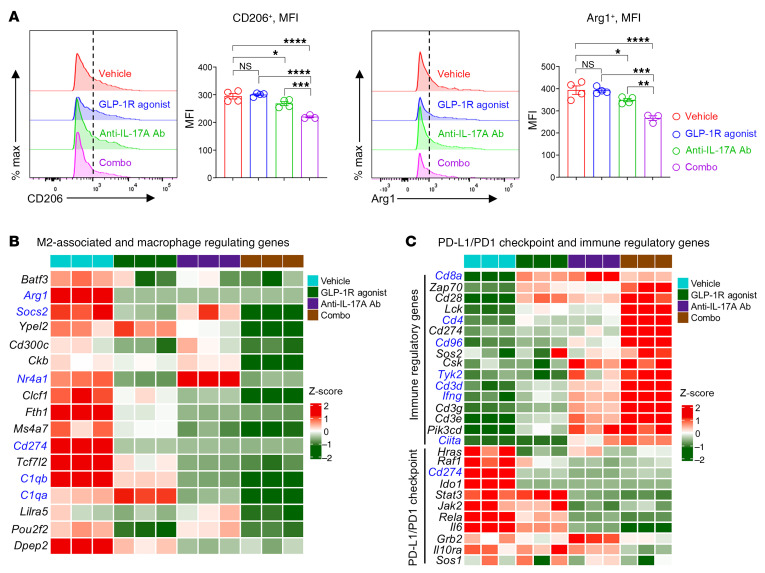
Combo therapy with a GLP-1R agonist and an anti–IL-17A antibody reduces M2-like macrophages in *Ob/Ob* mice bearing *Shp2^E76K/+^* HSC/Ps. (**A**) Flow cytometry plots and quantification of F4/80^+^CD206^+^ and F4/80^+^Arg1^+^ M2-like TAMs in adipose tissue following vehicle or Combo therapy. (**B**) Heatmap showing expression of M2-associated macrophage markers and macrophage-regulatory genes across treatment groups. (**C**) Heatmap displaying expression of the PD-1/PD-L1 checkpoint pathway and immune-regulatory genes across treatment groups. *n* = 3–4 mice per group. *P < 0.05, **P < 0.005, ***P < 0.0005, and ****P < 0.0001, by 1-way ANOVA followed by Tukey’s post hoc test. Data are presented as the mean ± SEM.

**Figure 13:**
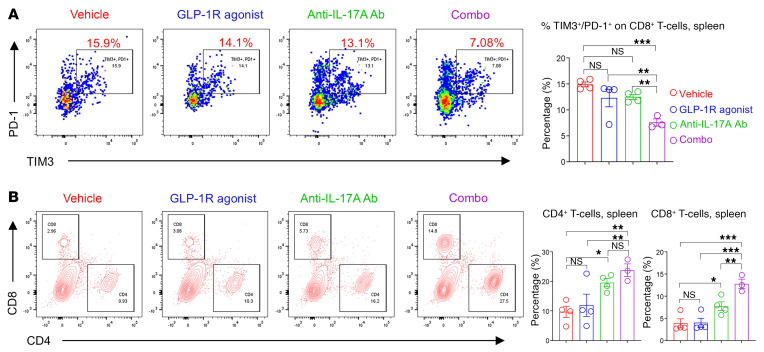
Combo therapy with a GLP-1R agonist and an anti–IL-17A antibody reduces T cell exhaustion and restore cytotoxic T cells in *Ob/Ob* mice bearing *Shp2^E76K/+^*-mutant HSC/Ps. (**A**) Flow cytometry plots and quantification of TIM3^+^PD-1^+^ exhausted T cells on CD8^+^ T cells in the spleens of *Ob/Ob* mice transplanted with *Shp2^E76K/+^* HSC/Ps following the indicated drug treatment. (**B**) Flow cytometry plots and quantification of CD4^+^ and CD8^+^ T cell populations in the spleens of *Ob/Ob* mice transplanted with *Shp2^E76K/+^* HSC/Ps following the indicated drug treatment. *n* = 3–4 mice per group, *P < 0.05, **P < 0.005, and ***P < 0.0005, by 1-way ANOVA followed by Tukey’s post hoc test. Data are presented as the mean ± SEM.

In line with the accumulation of M2-like TAMs observed in *Ob/Ob* recipients of *Shp2^E76K/+^*-mutant HSC/Ps (Figure 10H), Combo therapy with an anti–IL-17A antibody and a GLP-1R agonist markedly reduced CD206^+^ and Arg1^+^ M2-like TAMs compared with either monotherapy- or vehicle-treated mice (Figure 12A). Supporting this observation, RNA-seq analysis revealed consistent downregulation of M2-like TAMs associated and macrophage-regulatory gene expression following Combo treatment (Figure 12B). In *Ob/Ob* mice bearing *Shp2^E76K/+^*-mutant HSC/Ps, immune dysregulation was further characterized by elevated *Cd274* (*PDL1*) expression, together with reduced expression of class II major histocompatibility complex transactivator (*Ciita*), *Tyk2*, and *Cd96*. Increased *PDL1* expression on hematopoietic and immune cells enhances *PD1*-mediated inhibitory signaling, promoting T cell exhaustion and enabling tumor cells to evade immune surveillance (40,41). Meanwhile, reduced *Ciita* expression limits MHC class II–dependent antigen presentation, impairing CD4^+^ T cell priming (42); decreased *Tyk2* blunts IFN-γ signaling, weakening Th1 differentiation and NK cell activity (43); and loss of *Cd96* diminishes NK and T cell–mediated cytotoxicity (44). Treatment with either an anti–IL-17A antibody or a GLP-1R agonist alone produced only modest improvements in these defects. In contrast, Combo therapy showed reduced *Pdl1* expression, alleviating T cell exhaustion and restoring effective T cell responses (Figure 12C). Concurrently, recovery of *Ciita* enhanced antigen presentation, increased *Tyk2* expression, revitalized *IFNG*-dependent signaling to support Th1 and NK cell activity, and restored *Cd96* expression, further strengthening NK and T cell–mediated surveillance. Importantly, expression of *Cd3d*, *Cd4*, and *Cd8a* was also robustly upregulated with Combo therapy, indicating reactivation of TCR signaling and improved CD4^+^ helper and CD8^+^ cytotoxic T cell function (Figure 12C). Flow cytometric analysis confirmed our findings that treatment with either an anti–IL-17A antibody or a GLP-1R agonist alone resulted in modest improvements and that Combo-treated mice had reduced T cell exhaustion, as evidenced by decreased TIM3^+^PD-1^+^ coexpression (Figure 13A), and showed increased enrichment of both CD4^+^ and CD8^+^ T cell subsets (Figure 13B). Together, these results demonstrate that dual GLP-1R agonism and IL-17A blockade not only suppressed obesity- and *Shp2^E76K/+^*-driven macrophage expansion and inflammatory reprogramming but also relieved T cell exhaustion and restored cytotoxic T cell immunity. This dual targeting strategy, therefore, acted at the intersection of metabolic dysfunction, myeloid-driven inflammation, and immune suppression, providing a powerful therapeutic avenue for controlling myeloproliferation in the context of obesity and oncogenic SHP2 signaling.

## Discussion

Our integrative study elucidates a compelling link between obesity, metabolic dysfunction, and the pathogenesis of myeloid malignancies driven by oncogenic *PTPN11* mutations, advancing our understanding of how metabolic states influence hematopoietic transformation and leukemia progression. Utilizing genetically engineered mouse models, we demonstrate that obesity and diabetes potentiated the expansion and leukemogenic capacity of *Shp2^E76K/+^*-mutant HSC/Ps, driving a robust myeloproliferative neoplasm–like (MPN-like) phenotype. This was characterized by increased engraftment, myeloid hyperproliferation, hepatosplenomegaly, and elevated inflammatory cytokines, particularly IL-17A, and reduced GLP-1R expression. Importantly, dual targeting of IL-17A and metabolic pathways with a GLP-1R agonist synergistically suppressed mutant cell expansion, restored normal hematopoiesis, and mitigated obesity-driven inflammatory reprogramming in BM and adipose macrophages.

These preclinical insights are corroborated and extended by large-scale human epidemiological data from the UK Biobank and genomic databases. Our analysis of over 440,000 individuals revealed that, while BMI strongly predicts T2D, it is specifically the WHR, a measure of central adiposity and metabolic risk, that is associated with an increased incidence of myeloid leukemia, consistent with emerging evidence linking visceral fat and metabolic inflammation to hematologic malignancies (45, 46). The lack of a strong association between BMI and leukemia risk aligns with prior reports underscoring the importance of fat distribution rather than absolute adiposity (5).

Genetic analysis of *PTPN11* rare missense variants revealed associations with reduced lean mass and increased adiposity, implicating *PTPN11* mutations in the pathogenesis of obesity-related metabolic dysregulation. Notably, we observed enrichment of *PTPN11* mutations in patients with obesity who had cancer within TCGA cohorts, supporting a synergistic interplay between metabolic status and oncogenic signaling pathways. This complements murine data, in which obesity-induced inflammation fosters the expansion of *Ptpn11^E76K/+^*-mutant HSC/Ps, accelerating leukemogenesis.

Circulating IL-17A levels, a pivotal proinflammatory cytokine implicated in tumor progression and chronic inflammation (28, 47), were elevated in individuals with obesity, diabetes, or both, whereas GLP-1R levels were reduced in obesity, mirroring the dysregulated cytokine milieu observed in our obese mouse models. These systemic changes likely contribute to creating a proleukemic microenvironment by promoting myeloid cell expansion and hematopoietic niche remodeling. The successful preclinical use of IL-17A blockade and GLP-1R agonists to reverse hematopoietic and metabolic dysfunction highlights a promising translational avenue, underscored by ongoing clinical trials targeting these pathways in metabolic and inflammatory diseases (TOGETHER-PsO, NCT06588283).

Our transcriptomic profiling further delineates how obesity and *Ptpn11* mutation cooperate to remodel BM through activation of IL-17, TNF, and JAK/STAT signaling pathways, alongside metabolic rewiring involving lipid and cholesterol metabolism. This integrated inflammatory-metabolic reprogramming amplifies leukemogenic potential, consistent with emerging paradigms of cancer metabolism and immune dysregulation (48, 49). The marked attenuation of these pathways by combination therapy underscores the need for dual modulation of metabolic and inflammatory drivers in obesity-associated leukemia.

At the mechanistic level, our transcriptomic profiling of ATMs revealed that obesity and *Shp2^E76K/+^* mutations converged to drive both inflammatory and immunoregulatory macrophage programs, including IL-17, TNF, and JAK/STAT signaling, coupled with M2-like polarization and upregulation of checkpoint pathways such as the PD-1/PD-L1 pathway. These findings extend prior evidence implicating ATMs as key mediators of obesity-induced hematopoietic dysfunction (18, 50). Combo therapy with GLP-1R agonism and IL-17A blockade not only reduced overall F4/80^+^ ATM accumulation but also reversed the inflammatory transcriptional signatures of these cells, including downregulation of proinflammatory cytokine genes (*Il1b*, *Il6*, *Il17a*, *Il23a*) and M2-associated genes (*Arg1*, *Cd274*, *Chil3*, *Il10*). In line with these transcriptomic findings, flow cytometric analysis confirmed marked reductions in CD206^+^ and Arg1^+^ M2-like macrophages compared with either monotherapy or vehicle treatment. Thus, the therapy acted dually on macrophage quantity and quality, resetting their activation state toward a less inflammatory phenotype.

A key extension of our findings is that macrophage reprogramming was coupled to the restoration of adaptive immune function. Specifically, Combo therapy alleviated CD8^+^ T cell exhaustion marked by decreased TIM3^+^PD-1^+^ expression and reduced expression of PD-1/PD-L1 signaling genes. This immunomodulatory shift coincided with enrichment of cytotoxic T cell populations, including both CD4^+^ and CD8^+^ subsets, suggesting that therapy not only dampened myeloid-driven inflammation but also reinstated effective antileukemic immunity. Together, these results position the obesity *PTPN11* axis as a driver of a myeloid lymphoid crosstalk that fuels leukemogenesis, whereby macrophage expansion and immune checkpoint activation suppress effective T cell responses, and highlight how dual therapy dismantles this inflammatory-immune–suppressive circuit.

Collectively, our study provides mechanistic and clinical evidence linking obesity, metabolic inflammation, and *PTPN11*-driven hematopoietic malignancies. We establish that the IL-17A/GLP-1R axis orchestrated both macrophage-driven inflammation and T cell exhaustion, thereby creating a permissive microenvironment for leukemic progression. By showing that dual GLP-1R agonism and IL-17A blockade not only restrained myeloid proliferation but also restored immune competence, our work highlights a rational therapeutic strategy that targets the metabolic-immune axis. These findings advocate for precision medicine approaches that integrate metabolic modulation with immunotherapy to mitigate obesity-associated leukemia risk and progression.

## Methods

### Sex as a biological variable.

Our study examined male and female animals, and similar findings are reported for both sexes.

### Study population.

The UK Biobank is a large-scale cohort study that recruited approximately 500,000 individuals aged 40–69 years between 2006 and 2010 from 22 assessment centers across the United Kingdom. At enrollment, participants provided biological samples and detailed baseline clinical information. Longitudinal follow-up data were collected through medical history records, hospital admissions, and death registries. A comprehensive description of the UK Biobank has been published previously (51).

For the present study, we first included 441,874 unrelated participants from the UK Biobank who underwent exome sequencing from collected blood samples (52). The final analytical cohort comprised 441,469 unrelated and consented individuals with available exome sequencing data and measurements of either BMI or WHR, where unrelated individuals were defined as having no closer than third-degree kinship based on centrally calculated genomic relationship coefficients (52).

### Genotype processing.

Detailed methods for genotyping, processing, quality control, and imputation of the UK Biobank genetic data have been documented elsewhere (51, 52). In this analysis, genetic data were utilized solely for principal component analysis (PCA) to adjust for population stratification. PCA was then performed using fastPCA on a pruned set of 147,604 single-nucleotide variants among unrelated participants.

### Study outcomes.

The outcomes for this study were incidences of T2D and myeloid malignancies. Participants with prevalent diagnoses of these conditions at baseline were excluded from the respective analyses. Disease outcomes were ascertained through a combination of self-reported data, hospital inpatient records (Hospital Episode Statistics for England, Scottish Morbidity Records, and Patient Episode Database for Wales), and UK death registry data. Details of fields and diagnosis codes to define both phenotypes are described in Supplemental Table 3.

### Mice.

All mice used for these studies were a mix of male and female mice on a C57BL/6J background. The leptin-deficient *Lep^Ob/Ob^* (*Ob/Ob*) mice (stock no. 000632) and leptin receptor–deficient *Lepr^Db/Db^ (Db/Db)* mice (stock no. 000697) on a C57BL/6 background were purchased from The Jackson Laboratory, and C57BL/6 mice were procured from the IUSM core facility and used as WT controls. *Shp2^E76K/+^* *LysM-Cre^+^* mice have been described previously (53, 54) and were identified by genotyping genomic DNA from tail snips. All mice were bred and maintained under specific pathogen–free conditions in the animal facility at the IUSM with a 12-hour light/12-hour dark cycle and were provided food and water ad libitum.

### Competitive BMT.

Ten- to 12-week-old recipient animals (*Ob/Ob*, *Db/Db*, and WT) were lethally irradiated (700 cGy plus 400 cGy) 1 day prior to transplantation (via i.v. tail injection) of donor cells. The CD45.2^+^ donor BM cells from 20-week-old *Shp2^E76K/+^ LysM-Cre*^+^ mice were mixed with age-matched Boy/J CD45.1^+^ competitor BM donor cells (with an equal number of viable total cells, 500,000:500,000) and monitored for disease progression.

### In vivo drug treatment.

The CD45.2^+^ donor BM cells from *Shp2^E76K/+^ LysM-Cre*^+^ mice were mixed with age-matched Boy/J CD45.1^+^ competitor BM donor cells (with an equal number of viable total BM cells, 500,000:500,000). Recipient mice (*Ob/Ob* and WT) were lethally irradiated (700 cGy plus 400 cGy) 1 day prior to transplantation (via i.v. tail injection) of donor cells (55). Eight weeks after transplantation, the *Ob/Ob* recipient mice were then randomized and treated for 30 days with beinaglutide, a GLP-1R agonist (3 mg/kg, s.c.), an anti–IL-17A antibody (200 μg/mouse, i.p., 3 times weekly), or their combination. Dosing regimens were selected on the basis of prior studies demonstrating efficacy with minimal toxicity (38, 39).

### Flow cytometric analysis.

BM cells were harvested from femurs and tibias, and single-cell suspensions were stained with fluorophore-conjugated antibodies for lineage and stem/progenitor markers. Flow cytometry was performed as described previously (56). Multiparameter flow cytometric analysis was performed using an LSRFortessa flow cytometer with Diva software (BD Biosciences), and the data were analyzed using FlowJo software (version 10.7.0) (55–57). The flow cytometry markers used are described in the Supplemental Methods (Supplemental Table 4).

Details of the materials and methods used in this study are provided in the Supplemental Methods.

### Statistics.

Data are presented as the mean ± SEM for all mice analyzed in this study. One-way ANOVA followed by Tukey’s post hoc test for multiple comparisons and 2-tailed Student’s *t* test between 2 groups were used to evaluate statistical significance. A *P* value of less than 0.05 was considered statistically significant.

### Study approval.

The mouse studies were approved by the Indiana University Laboratory Animal Resource Center, and all experiments were conducted at the Laboratory Animal Resource Center according to the protocol.

### Data availability.

Data can be found in the Supporting Data Values file. Data on the cytokines and chemokines analyzed in this study were from patients with AML and were obtained from the Gene Expression Omnibus (GEO) database microarray dataset GSE1159. Mouse RNA-seq data analyzed in this study have been deposited in the GEO database (GEO GSE327968).

## Author contributions

R Kapur, LL, and R Kanumuri contributed equally as co–first authors. The authorship order among the co–first authors was determined on the basis of the extent of their overall contributions to the study. R Kapur, LL, and R Kanumuri designed and performed the experiments and analyzed the data. SKP and ZY designed and executed the experiments and analyzed the data. R Kanumuri, BR, LRP, and R Kumar assisted with the experiments. KSRP analyzed the RNA-seq data and contributed to data interpretation and figure preparation. CP and GC helped with the *PTPN11* structural analysis. ZY, PN, SK, and LL provided and analyzed human exome sequencing data from the UK Biobank. LSH read the manuscript and provided critical input. SKP, LL, ZY, and R Kapur wrote the manuscript. All authors read and approved the manuscript.

## Conflict of interest

PN reports research grants from Allelica, Amgen, Apple, Boston Scientific, Cleerly, Genentech/Roche, Ionis, Novartis, and Silence Therapeutics; personal fees from AIRNA, Allelica, Apple, AstraZeneca, Bain Capital, Blackstone Life Sciences, Bristol Myers Squibb, Creative Education Concepts, CRISPR Therapeutics, Eli Lilly & Co., Esperion Therapeutics, Foresite Capital, Foresite Labs, Genentech/Roche, GV, HeartFlow, Incyte, Magnet Biomedicine, Merck, Novartis, Novo Nordisk, TenSixteen Bio, and Tourmaline Bio; has equity in Bolt, Candela, Mercury, MyOme, Parameter Health, Preciseli, and TenSixteen Bio; receives royalties from Recora for intensive cardiac rehabilitation; and spousal employment at Vertex Pharmaceuticals.

## Funding support

This work is the result of NIH funding, in whole or in part, and is subject to the NIH Public Access Policy. Through acceptance of this federal funding, the NIH has been given a right to make the work publicly available in PubMed Central.

- Herman B Wells Center and the Riley Children’s Foundation (to SKP).
- Ralph W. and Grace M. Showalter Research Trust and the Indiana University School of Medicine (to SKP).
- Developmental and Hyperactive Ras Tumor (DHART) SPORE Developmental Research Program funded by the National Cancer Institute (NCI), NIH (U54CA196519, to SKP).
- Leukemia Research Foundation (grant 1306942, to SKP).
- Alex’s Lemonade Stand Foundation (grant 1349722, to SKP).
- NIH (R01CA173852, R01CA134777, R01HL146137, and R01HL140961, to R Kapur).
- Riley Children’s Foundation (to R Kapur).
- NIH (R01HL168894, to PN).
- UK Biobank (application 7089, to PN).

## Supplementary Material

Supplemental data

Supplemental data set 1

Supporting data values

## Figures and Tables

**Table 6:**
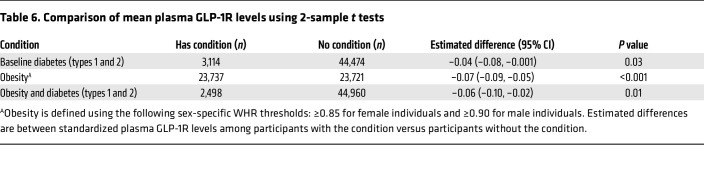
Comparison of mean plasma GLP-1R levels using 2-sample *t* tests

**Table 5:**
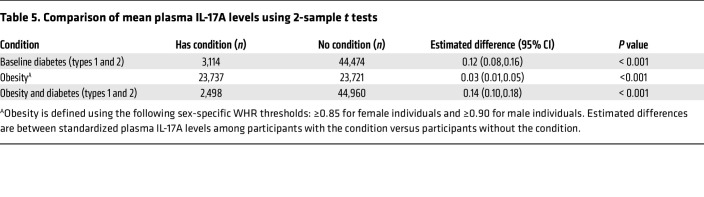
Comparison of mean plasma IL-17A levels using 2-sample *t* tests

**Table 4:**
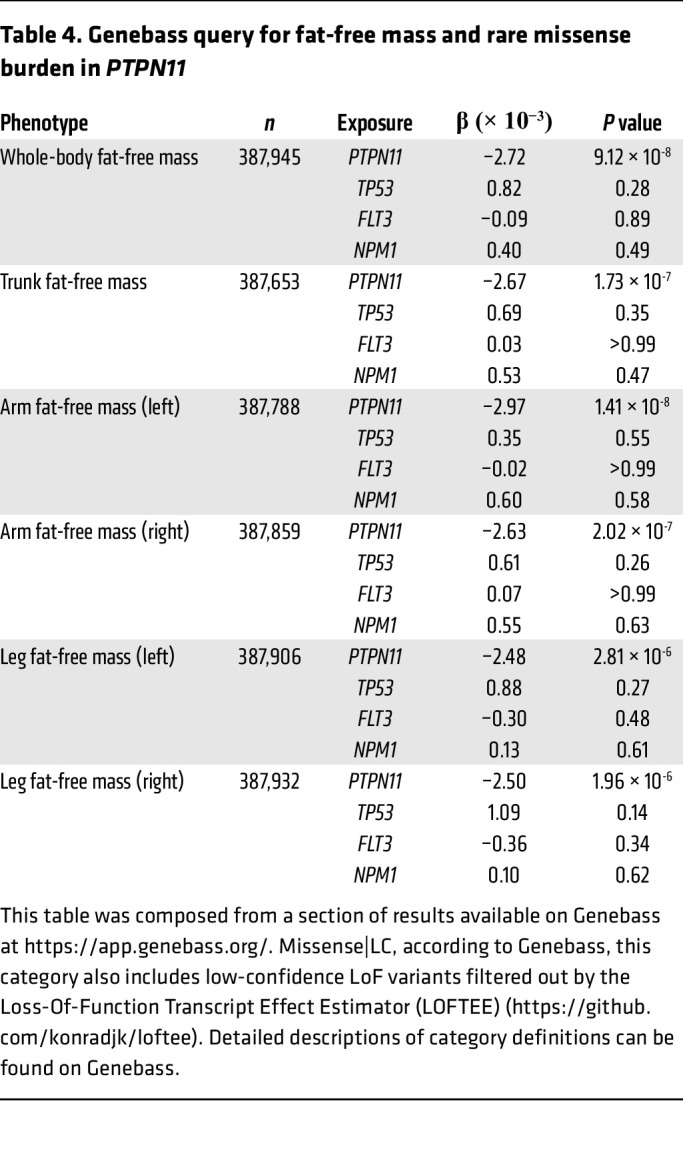
Genebass query for fat-free mass and rare missense burden in *PTPN11*

**Table 3:**
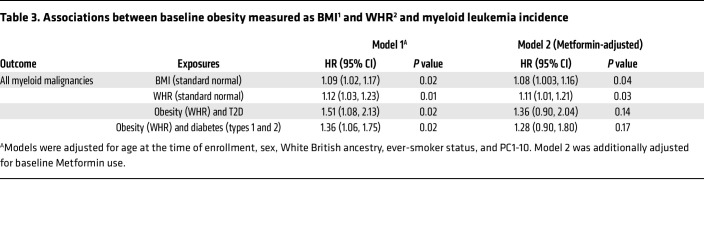
Associations between baseline obesity measured as BMI^1^ and WHR^2^ and myeloid leukemia incidence

**Table 2:**
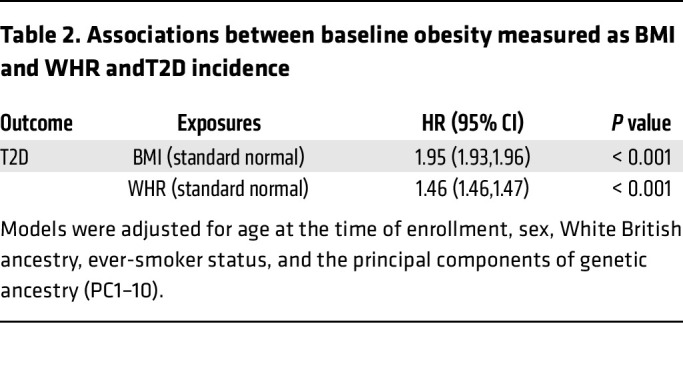
Associations between baseline obesity measured as BMI and WHR andT2D incidence

**Table 1:**
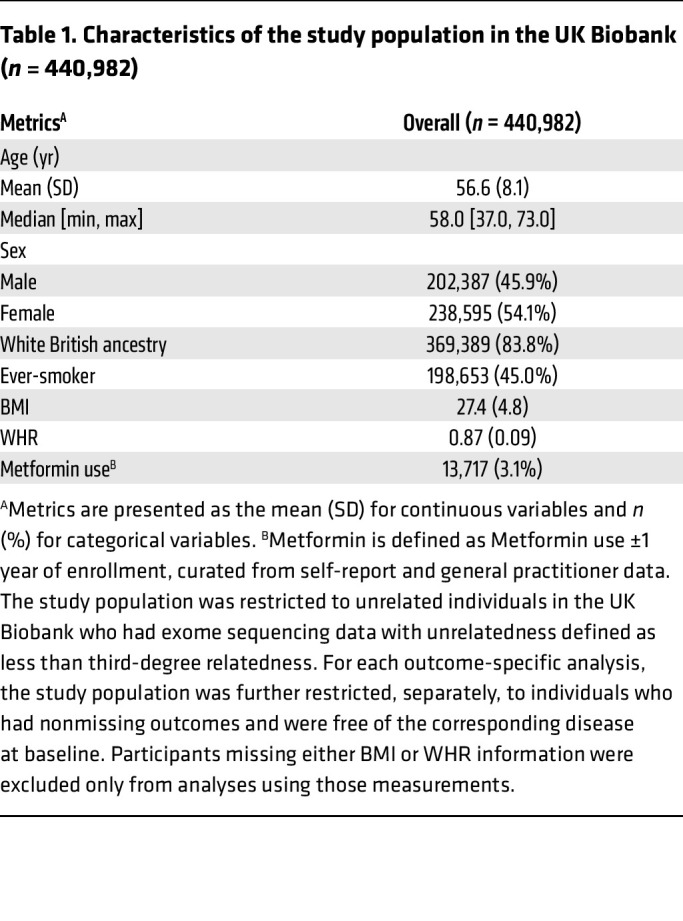
Characteristics of the study population in the UK Biobank (*n* = 440,982)

## Version 1 06/23/2026

In-Press Preview

## Version 2 08/03/2026

Electronic publication
