# Supplementary material for: Targeting GLP-1R and IL-17A suppresses obesity-induced leukemia in an oncogenic PTPN11 mutation–driven model
Source: J Clin Invest. 2026 Jun 23;136(15):e202856. doi: 10.1172/JCI202856 (PMC13430024; doi:10.1172/JCI202856)
Supplement: Supplemental data [file jci-136-202856-s181.pdf]

Targeting GLP1R and IL17A suppresses obesity-induced leukemia in an oncogenic *PTPN11* mutation-driven model

Reuben Kapur[1,&,*], Linke Li[2,3,*], Rahul Kanumuri[1,*], Kanaka Sai Ram Padam[1], Baskar Ramdas[1], Chiranjeevi Pasala[4], Gabriela Chiosis[4], Lakshmi Reddy Palam[1], Ramesh Kumar[1], Satoshi Koyama[2,3], Pradeep Natarajan[3,5,6], Laura S. Haneline[1,&], Zhi Yu[2,3,6,&,#], and Santhosh Kumar Pasupuleti[1,&,#]

Supplemental methods

Rare variants analysis

We utilized Genebass (https://app.genebass.org/) (1), a publicly available resource providing gene-based association results from 394,841 UK Biobank exomes, generated using SAIGE-GENE (1). Genebass offers results from various rare variant analysis methods, including burden tests, sequence kernel association tests (SKAT), and SKAT-O (optimal unified SKAT tests), for thousands of UK Biobank native and custom-derived phenotypes. We specifically queried gene-based burden test results for rare (MAF < 0.1%) missense variants in *PTPN11* against fat-free mass body composition measures by impedance (Category 100009). These continuous phenotypes were inverse rank normal transformed as part of the standard Genebass processing workflow. Fat-free mass was selected as a proxy for lean mass, aligning with our mouse models.

Baseline Metformin use

Metformin usage information was curated from both the self-report (SR) data (field 20003) and the general practitioner (GP) data using matched drug names (Glucophage, Metformin, Axpnet, Diagemet, Glucient, Metabet, Glumetza, Fortamet, Riomet). Baseline metformin use was defined

as any recorded use in either data source within ±1 year of the participant's baseline enrollment date.

Circulating IL-17A and GLP-1R measurement

Plasma IL-17A and GLP-1R protein levels were measured as part of a large-scale proteomic assessment in a subset of approximately 50,000 UK Biobank participants. Details of the Olin Target 96-plex protein biomarker assay and quality control procedures have been described previously by the UK Biobank (2). For this analysis, IL-17A and GLP-1R levels were standardized (mean 0, SD 1) prior to the analysis.

Statistical analysis

Cox's proportional-hazard models (R package survival, version 3.1.12) were employed to examine the association of standardized BMI and standardized WHR with the risks of incident T2D and myeloid leukemia. Follow-up time was calculated from the date of enrollment to the date of first diagnosis of the outcome, death, loss to follow-up, or the end of the study period, whichever occurred first. All models were adjusted for age at enrollment (continuous), sex (male/female), ever-smoker status (yes/no), and the first 10 principal components of genetic ancestry. Given the reported protective effect of metformin and acute myeloid leukemia (3–6), a sensitivity analysis further adjusting for baseline metformin use was performed when examining the association between BMI and WHR with the incidence of myeloid leukemia.

Two-sample t-tests were used to compare the differences in mean standardized plasma IL-17A and GLP-1R level between groups defined by baseline diabetes status and obesity status. We defined obesity as a WHR ≥0.85 in female participants and ≥0.90 in male participants. Statistical significance was determined using two-tailed p-values, with a threshold of $P < 0.05$. All statistical analyses were performed using R version 4.0.0.

Peripheral blood counts

Total white blood cell counts in freshly collected mouse blood were performed using Element HT5 Hematology Analyzer (Heska).

Serum cytokine and chemokine analysis

Serum was separated from peripheral blood and subjected to mouse serum cytokine/chemokine (array MD31-plex) analysis through Eve Technologies (Canada). Results are expressed as pg/mL of serum (7,8).

RNA sequencing and differential gene expression analysis

RNA was extracted from BM and ATMs of drug-treated *Ob/Ob* mice bearing *Shp2*$^{E76K/+}$ cells using RLT lysis buffer and the RNeasy Plus Micro Kit (Qiagen), following the manufacturer's instructions. RNA samples were processed according to standard protocols, and sequencing was performed at the Indiana University Genomics Core Facility. RNA-seq reads were aligned to the mouse reference genome (mm10) using STAR (v2.7.2a) with the parameter "--outSAMmapqUnique 60" (9). Uniquely mapped reads were quantified against GENCODE M22 gene annotations using featureCounts (v2.0.1) with the parameters "-s 0 -p -Q 10 -O" (10).

The mapped raw read counts were corrected for batch effects and library preparation using DESeq2 (11) R package in R program (R Core Team (2020). R: A language and environment for statistical computing. R for Statistical Computing, Vienna, Austria. URL https://www.R-project.org/). The differential expression analysis was performed by setting a threshold of log2FC (<-1 and >+1) with p<0.05 to control the false positives at 5% false discovery rate (FDR). Further, the gene sets of specific biological queries were visualized as heatmap using Complex Heatmap (12) package.

Functional enrichment analysis

Gene set annotation and enrichment analysis of the differentially expressed genes were analyzed using the gene ontology (GO) biological processes (BP), molecular function (MF) and the Kyoto encyclopedia of the genes and genomes (KEGG) pathways using the ClusterProfiler (13) R package. An adjusted p-value (<0.05) with the Bonferroni-Hochberg correction was applied to determine the statistical significance between the query set and the mapped hits. Furthermore, the cancer hallmark gene sets summarizing the biological states and cell type specific signatures were retrieved from the molecular signatures database (MSigDB) collection, analyzed using msigdbr R package.

Deconvolution analysis

The murine Microenvironment Cell Population (mMCP) counter analysis was performed for deconvolution of bone marrow bulk RNA-seq data to estimate the relative abundance of the various immune cell populations. The pre-defined marker genes expression per cell type population were accessed from the immunological genome project (ImmGen) using the immunedeconv (14) and mMCP (15) R packages. One-way analysis of variance (ANOVA) was used to determine the statistical significance using the Graph Pad Prism (v10, Graph Pad Software Inc., USA).

Polymorphism Phenotyping (PolyPhen) and Sift score calculation

The Bioinformatics tools like PolyPhen-2 (Polymorphism Phenotyping V2) (16) and the Sift (Sorting Intolerant From Tolerant) score (17) online web servers were used to predict the impact of the stability and function of *PTPN11* gene mutations.

References:

1. Karczewski KJ, Solomonson M, Chao KR, Goodrich JK, Tiao G, Lu W, et al. Systematic single-variant and gene-based association testing of thousands of phenotypes in 394,841 UK Biobank exomes. Cell genomics. 2022 Sep;2(9):100168.

2. Sun BB, Chiou J, Traylor M, Benner C, Hsu YH, Richardson TG, et al. Plasma proteomic associations with genetics and health in the UK Biobank. Nature. 2023;622(7982):329–38.

3. Gozdecka M, Dudek M, Wen S, Gu M, Stopforth RJ, Rak J, et al. Mitochondrial metabolism sustains DNMT3A-R882-mutant clonal haematopoiesis. Nature. 2025 Jun;642(8067):431–41.

4. Green AS, Chapuis N, Maciel TT, Willems L, Lambert M, Arnoult C, et al. The LKB1/AMPK signaling pathway has tumor suppressor activity in acute myeloid leukemia through the repression of mTOR-dependent oncogenic mRNA translation. Blood. 2010 Nov;116(20):4262–73.

5. Liu X, Chhipa RR, Pooya S, Wortman M, Yachyshin S, Chow LML, et al. Discrete mechanisms of mTOR and cell cycle regulation by AMPK agonists independent of AMPK. Proc Natl Acad Sci U S A. 2014 Jan;111(4):E435-44.

6. Saito T, Itoh M, Tohda S. Metformin suppresses the growth of leukemia cells partly through downregulation of AXL receptor tyrosine kinase. Leuk Res. 2020 Jul;94:106383.

7. Pasupuleti SK, Ramdas B, Burns SS, Palam LR, Kanumuri R, Kumar R, et al. Obesity-induced inflammation exacerbates clonal hematopoiesis. J Clin Invest. 2023 Jun;133(11).

8. Ramdas B, Mali RS, Palam LR, Pandey R, Cai Z, Pasupuleti SK, et al. Driver Mutations in Leukemia Promote Disease Pathogenesis through a Combination of Cell-Autonomous and Niche Modulation. Stem Cell Reports. 2020 Jul 14;15(1):95–109.

9. Dobin A, Davis CA, Schlesinger F, Drenkow J, Zaleski C, Jha S, et al. STAR: ultrafast universal RNA-seq aligner. Bioinformatics. 2013 Jan;29(1):15–21.

10. Liao Y, Smyth GK, Shi W. featureCounts: an efficient general purpose program for

assigning sequence reads to genomic features. Bioinformatics. 2014 Apr;30(7):923–30.

11. Love MI, Huber W, Anders S. Moderated estimation of fold change and dispersion for RNA-seq data with DESeq2. Genome Biol. 2014;15(12):1–21.

12. Gu Z. Complex heatmap visualization. iMeta. 2022 Sep;1(3):e43.

13. Yu G, Wang LG, Han Y, He QY. clusterProfiler: an R package for comparing biological themes among gene clusters. OMICS. 2012 May;16(5):284–7.

14. Sturm G, Finotello F, Petitprez F, Zhang JD, Baumbach J, Fridman WH, et al. Comprehensive evaluation of transcriptome-based cell-type quantification methods for immuno-oncology. Bioinformatics. 2019 Jul;35(14):i436–45.

15. Petitprez F, Levy S, Sun CM, Meylan M, Linhard C, Becht E, et al. The murine Microenvironment Cell Population counter method to estimate abundance of tissue-infiltrating immune and stromal cell populations in murine samples using gene expression. Genome Med. 2020;12(1):86.

16. Adzhubei I, Jordan DM, Sunyaev SR. Predicting functional effect of human missense mutations using PolyPhen-2. Curr Protoc Hum Genet. 2013 Jan;Chapter 7:Unit7.20.

17. Ng PC, Henikoff S. SIFT: Predicting amino acid changes that affect protein function. Nucleic Acids Res. 2003 Jul;31(13):3812–4.

Supplemental Figures:

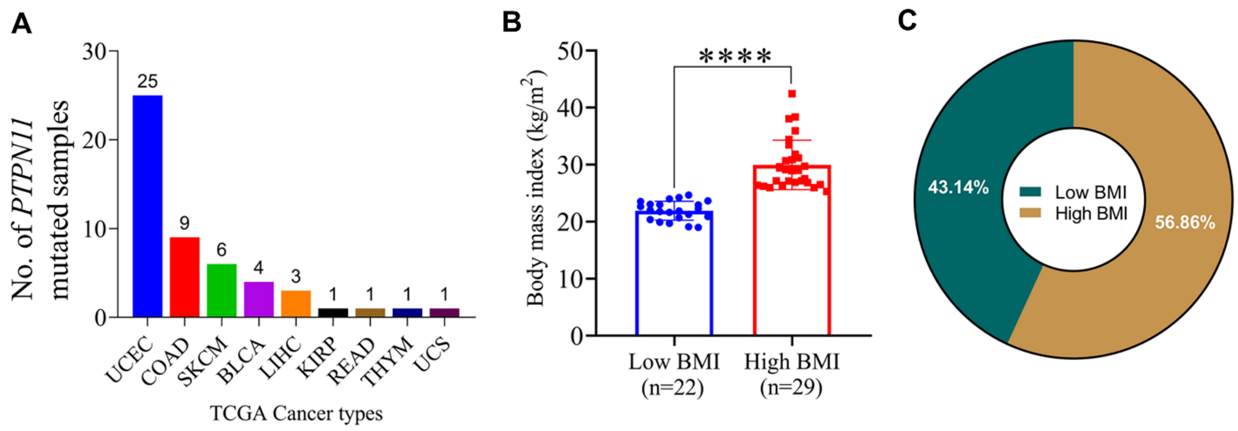

Supplemental Figure 1: Association of *PTPN11* mutations with obesity-related traits and cancer prevalence.

(**A)** Bar graph showing the number of *PTPN11*-mutated samples across various cancer types in The Cancer Genome Atlas (TCGA) cohort. Uterine corpus endometrial carcinoma (UCEC) exhibits the highest frequency. **B)** Comparison of BMI between patients with low (<25 kg/m^2) and high (>30 kg/m^2) BMI stratified by *PTPN11* mutation status, showing significantly higher BMI in the high BMI group (****p < 0.0001, by Two-tailed unpaired t-test with Welch's correction). **C)** Pie chart illustrates the proportion of *PTPN11*-mutated cancer cases stratified by BMI category, revealing that 56.86% of mutations occur in patients with high BMI.

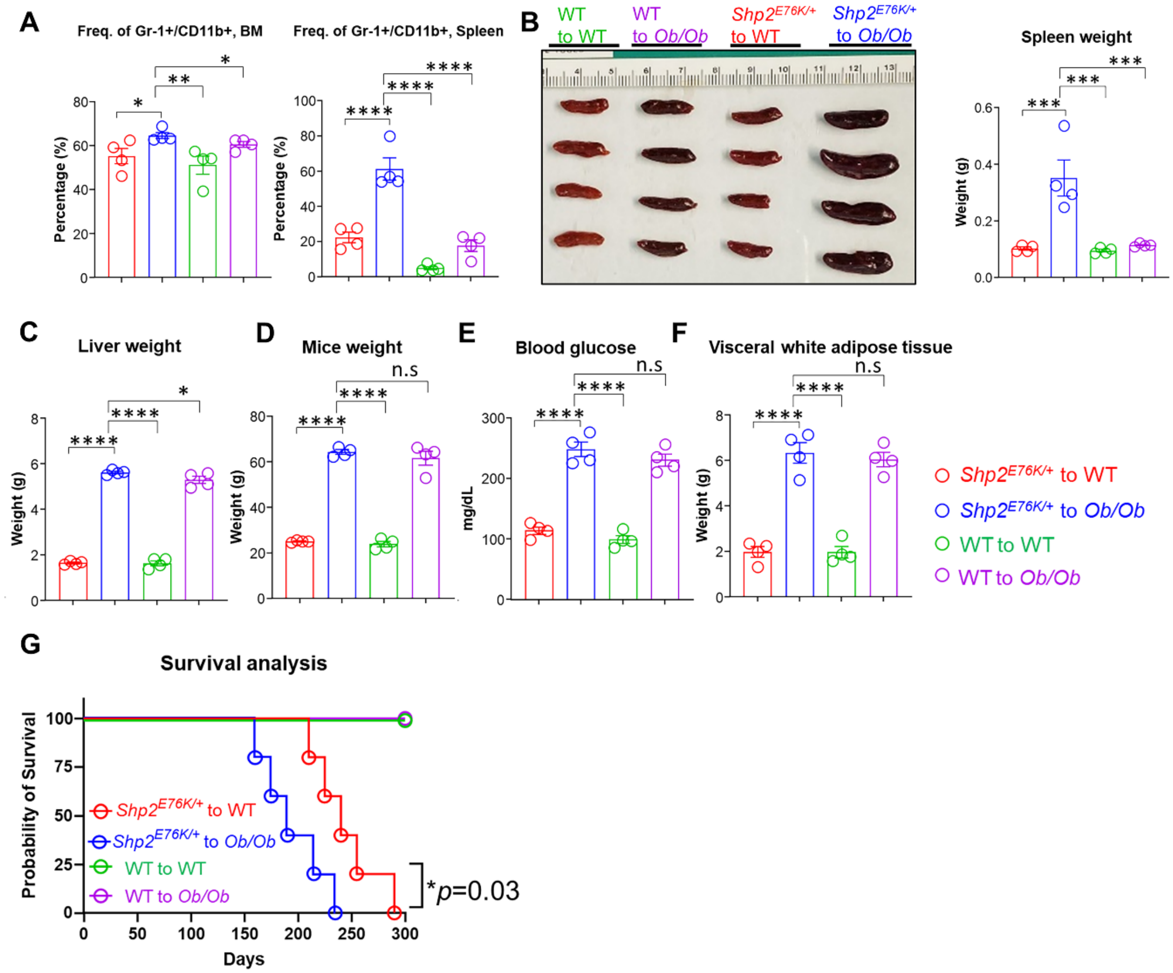

Supplemental Figure 2: *Shp2$^{E76K/+}$* mutant cells outcompete the normal WT cells in *Ob/Ob* recipients.

(A) Frequency of Gr-1^{+}/CD11b^{+} myeloid cells in BM and spleens of competitive transplant recipients over 24 weeks. **(B)** spleen pictures, right quantification of spleen weights, (**C)** mice body weights, **(D)** liver weights, **(E)** fasting blood glucose levels, and **(F)** visceral white adipose tissue weights. **(G)** Kaplan-Meier survival analysis of transplanted mice, showing reduced survival in *Ob/Ob* mice bearing *Shp2$^{E76K/+}$* cells compared to controls. n= 4 mice per group, **p<0.05, **p<0.005, ***p<0.0005,* and *****p<0.0001*, by one-way ANOVA followed by Tukey's post hoc test.

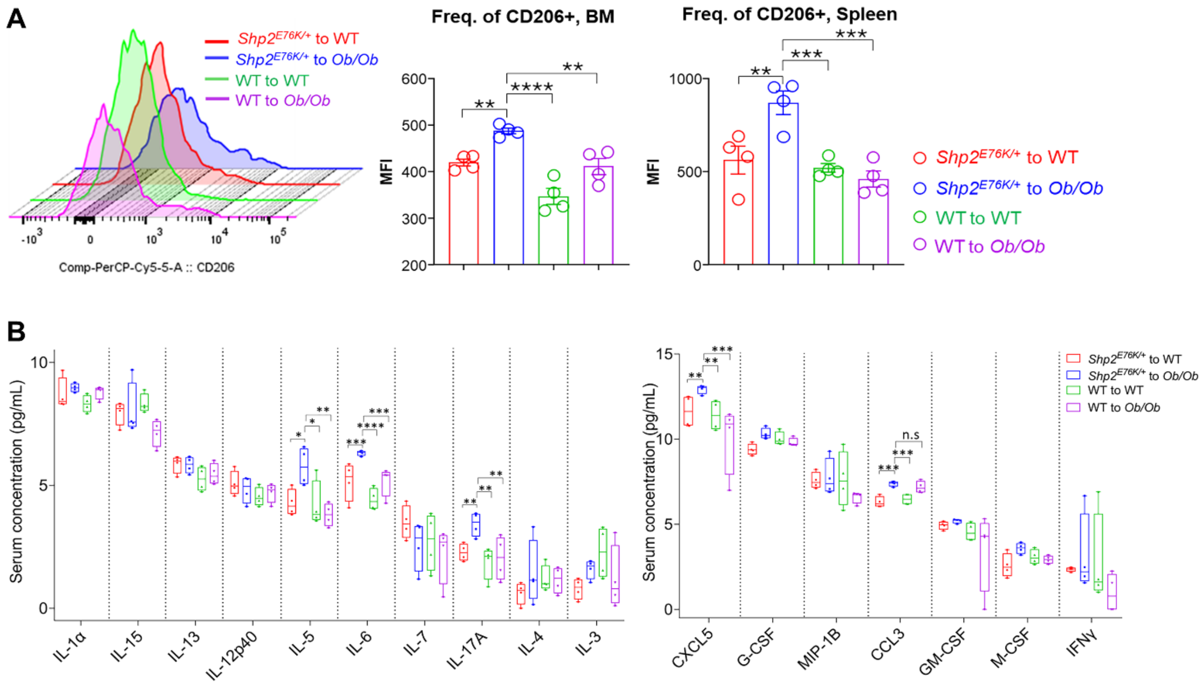

Supplemental Figure 3: ***Shp2*$^{E76K/+}$ mutant myeloid blasts and HSC/Ps expanded in *Ob/Ob* recipients.**

(A) Representative flow cytometry profile of F4/80+/CD206+ TAMs in the BM, right, frequency of F4/80+/CD206+ TAMs in the BM and spleens of competitive transplant recipients over 24 weeks. **(B)** Serum cytokine and chemokine analysis of recipient mice at 24 weeks after BMT. n= 4 mice per group, ***p<0.05,* ***p<0.005,* ****p<0.0005,* and *****p<0.0001*, by one-way ANOVA followed by Tukey's post hoc test.

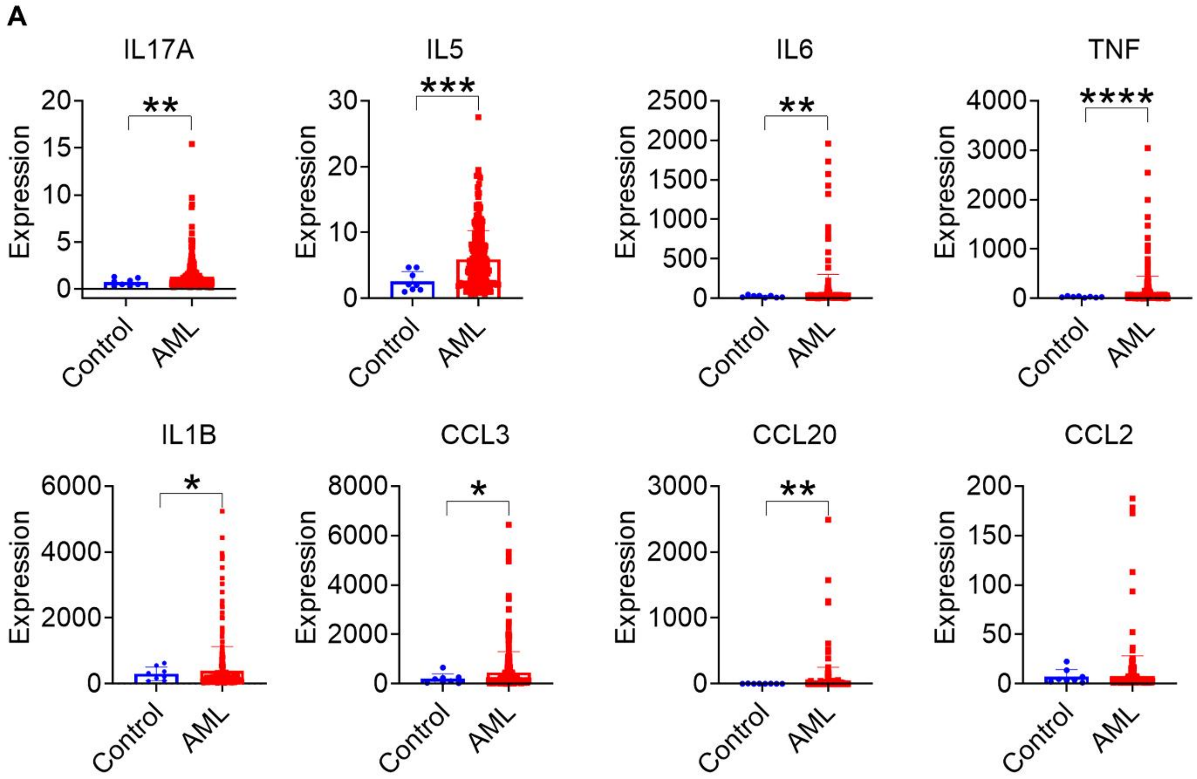

Supplementary Figure 4. Increased inflammatory cytokine and chemokine expression in AML patients compared to controls. Dot plots showing gene expression levels of IL17A, IL5, IL6, TNF, IL1B, CCL3, CCL20, and CCL2 in control (blue) and AML (red) samples. Each dot represents an individual sample. Controls (n= 8) and AML (n=285) analyzed using microarray dataset (GEO: GSE1159). *p<0.05, **p<0.005, ***p<0.0005, and ****p<0.0001, by Two-tailed unpaired t-test with Welch's correction.

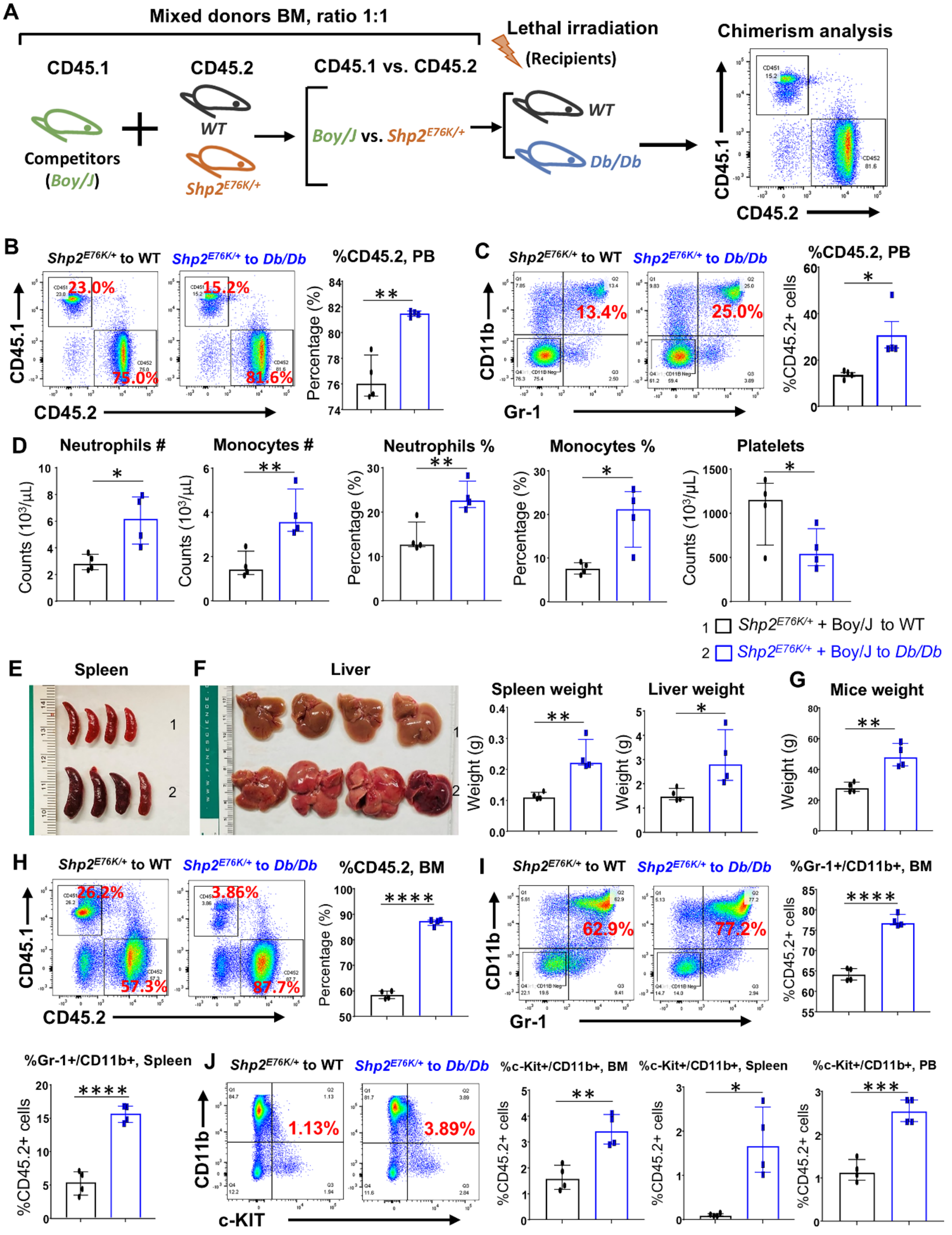

Supplemental Figure 5: *Shp2*$^{E76K/+}$ mutant cells outcompete the normal WT cells in *Db/Db* recipients. (A) Schematic of the competitive BMT assay. Donor BM cells from *Shp2*$^{E76K/+}$ mice

($CD45.2^+$) were mixed with Boy/J competitor ($CD45.1^+$) BM cells (500K:500K) were transplanted into lethally irradiated WT or *Db/Db* mice as recipients. Donor-derived chimerism was observed using anti-CD45.1 and anti-CD45.2 antibodies. **(B)** Representative profiles of flow cytometry for donor chimerism in the PB of recipient mice, (i) frequency of $CD45.2^+$ in the PB of indicated recipient mice. **(C)** Representative flow cytometry profile of $Gr\text{-}1^+/CD11b^+$ myeloid cells in the PB of the indicated recipient mice, (i) frequency of $Gr\text{-}1^+/CD11b^+$ myeloid cells in PB of competitive transplant recipients over 22 weeks. **(D)** Spleen and **(E)** liver pictures and their weights, **(F)** mice body weights. (**G)** PB counts at 22 weeks after BMT from the indicated recipient mice. **(H)** Representative flow cytometry profile of donor chimerism in the BM of recipient mice and **(I)** myeloid cells ($Gr\text{-}1^+/CD11b^+$) in the BM of the indicated recipient mice, (i) frequency of $Gr\text{-}1^+/CD11b^+$ myeloid cells in BM and (ii) spleens of competitive transplant recipients over 22 weeks. **(J)** Representative flow cytometry profiles of $c\text{-}KIT^+/CD11b^+$ myeloid blasts from the indicated recipient mice, (i) frequency of $c\text{-}KIT^+/CD11b^+$ cells in the BM, (ii) spleens and (iii) PB of competitive transplant recipients over 22 weeks. n= 4 mice per group, *$p<0.05$, **$p<0.005$, ***$p<0.0005$, ****$p<0.0001$, by Two-tailed unpaired t-test.

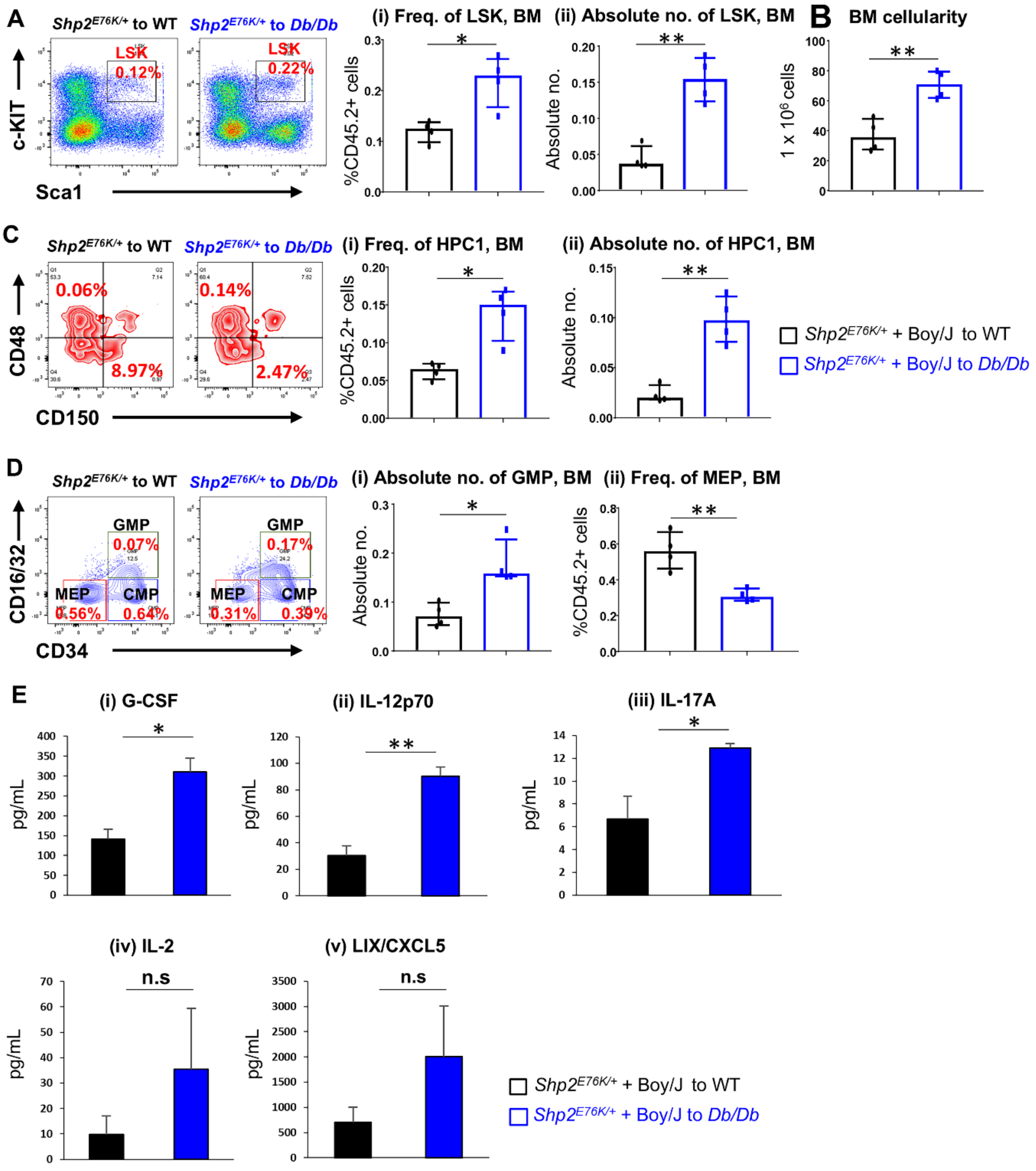

Supplemental Figure 6: *Shp2$^{E76K/+}$* mutant HSC/Ps expanded in *Db/Db* recipients. (A) Representative flow cytometry profile of LSK (Lin^{-}/Sca-1^{+}/c-KIT^{+}) cells in the BM from the indicated recipient mice, (i) frequency and (ii) absolute number of LSK cells in the BM and **(B)** BM cellularity of competitive transplant recipients over 22 weeks. **(C)** Representative flow cytometry profile of HPC1 (LSK/CD48^{+}/CD150^{-}) and LT-HSCs (LSK/CD48^{-}/CD150^{+}) in the BM from the

indicated recipient mice, (i) frequency and (ii) absolute number of HPC1 cells in the BM of competitive transplant recipients over 22 weeks. **(D)** Representative flow cytometry profile of progenitors in the BM, (i) absolute number of GMPs (Lin^{-}/c-KIT^{+}/CD16/32^{+}/$CD34^{+}$) in the BM of competitive transplant recipients over 22 weeks. **(E)** Serum cytokine and chemokine analysis, (i) G-CSF, (ii) IL-12p70, (iii) IL-17, (iv) IL-2, and (v) CXCL5 concentrations in the serum of recipient mice at 22 weeks after BMT. n= 4 mice per group, *p<0.05, **p<0.005, ***p<0.0005, ****p<0.0001, by Two-tailed unpaired t-test.

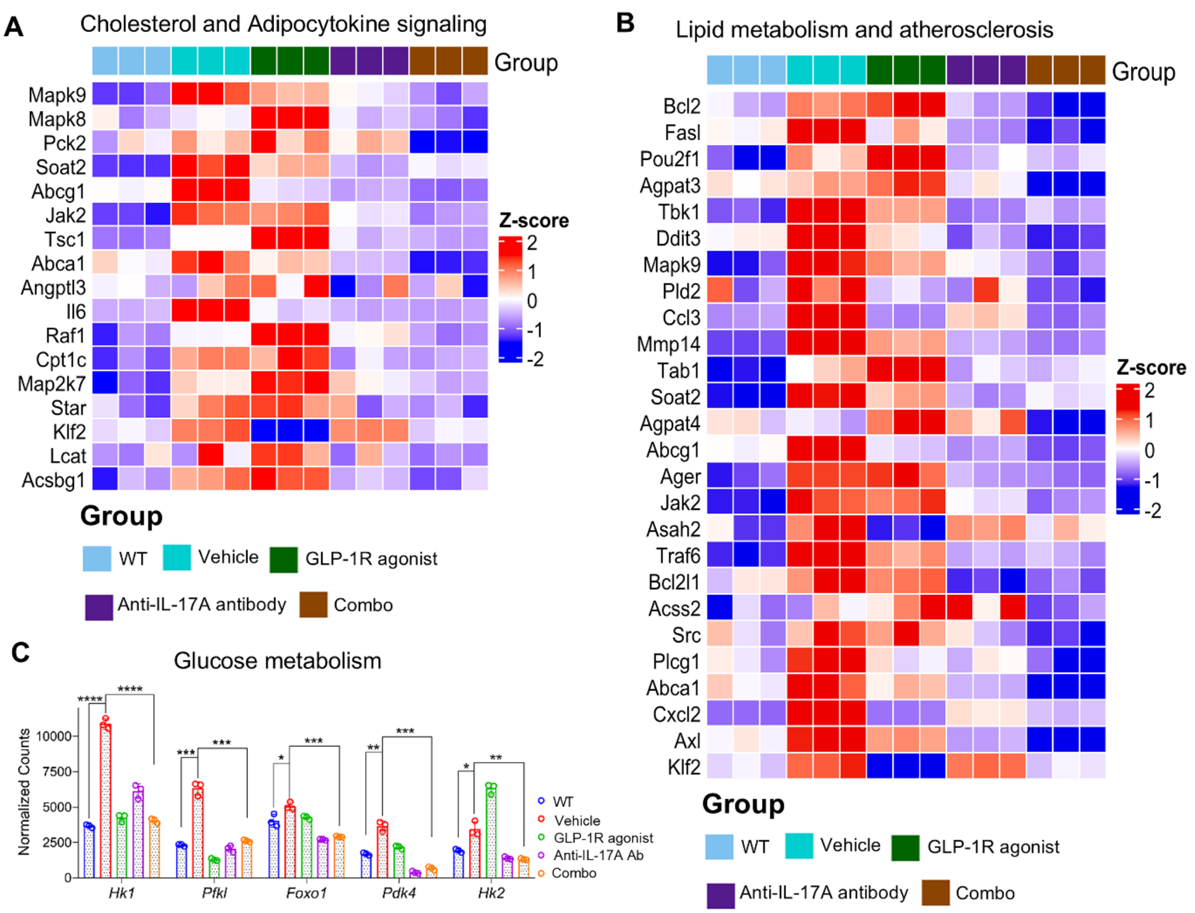

Supplemental Figure 7: Combination therapy with GLP-1R agonist and anti-IL-17A antibody reverses metabolic pathways in *Ob/Ob* mice bearing *Shp2*$^{E76K/+}$ HSC/Ps. (A and B) Metabolic pathway enrichment analysis with heatmap visualization of gene expression changes involved in **(A)** lipid metabolism and atherosclerosis, and **(b)** adipocytokine signaling, and cholesterol metabolism in indicated drug treatments. Expression data are represented as Z-scores normalized across groups. **(C)** Expression of glucose metabolism genes (*Hk1*, *Pfkl*, *Foxo1*, *Pdk4* and *Hk2*) in ATM derived from WT and *Ob/Ob* mice bearing *Shp2*$^{E76K/+}$ mice and treated with indicated drugs. *p<0.05, **p<0.005, ***p<0.0005, ****p<0.0001, by one-way ANOVA followed by Tukey's post hoc test.

Supplemental Table 1: *PTPN11* gene burden associations with overweight and BMI.

***PTPN11* gene burden associations with overweight**				
**Category**	**P-value SKATO**	**P-value burden**	**P-value SKAT**	**Total variants**
pLOF	0.0871	0.0517	0.298	8
Missense, LC	3.54E-07	3.29E-07	0.02	194
Synonymous	0.614	0.394	0.489	105
***PTPN11* gene burden associations with Body mass index (BMI)**				
**Category**	**P-value SKATO**	**P-value burden**	**P-value SKAT**	**Total variants**
pLOF	0.0237	0.0144	0.135	8
Missense, LC	3.03E-03	1.93E-03	0.0308	194
Synonymous	0.391	0.232	0.427	105

SKAT: Sequence kernel association tests.

SKAT-O: Sequence kernel association tests-optimal.

Supplemental Table 2: SIFT and PolyPhen-2 Predictions of Variant Pathogenicity.

**Amino acid change in *PTPN11***	**Sift score (Tolerance index)**	**PolyPhen-2 score**	**Sensitivity**	**Specificity**
E76K	0	0.998	0.27	0.99
E76D	0	0.948	0.79	0.95
V432M	0.01	1	0	1
P565L	0.24	0.985	0.74	0.96
M508V	0.03	1	0	1
N308D	0.01	0.625	0.87	0.91

Supplemental Table 3: Fields and Codes used in Phenotype curations.

Label	Field	Coding
Myeloid Malignancies	41202	C92,C92.0,C92.1,C92.2,C92.3,C92.4,C92.5,C92.6,C92.7,C92.8, C92.9,C93.0,C93.1,C93.2,C93.3,C93.7,C93.9,C94,C94.0,C94.1, C94.2,C94.3,C94.4,C96.0,C96.1,C96.2,C96.3,C96.4,C96.5,C96.6
	41204	C92,C92.0,C92.1,C92.2,C92.3,C92.4,C92.5,C92.6,C92.7,C92.8, C92.9,C93.0,C93.1,C93.2,C93.3,C93.7,C93.9,C94,C94.0,C94.1, C94.2,C94.3,C94.4,C96.0,C96.1,C96.2,C96.3,C96.4,C96.5,C96.6
	40001	C92,C92.0,C92.1,C92.2,C92.3,C92.4,C92.5,C92.6,C92.7,C92.8, C92.9,C93.0,C93.1,C93.2,C93.3,C93.7,C93.9,C94,C94.0,C94.1, C94.2,C94.3,C94.4,C96.0,C96.1,C96.2,C96.3,C96.4,C96.5,C96.6
	40002	C92,C92.0,C92.1,C92.2,C92.3,C92.4,C92.5,C92.6,C92.7,C92.8, C92.9,C93.0,C93.1,C93.2,C93.3,C93.7,C93.9,C94,C94.0,C94.1, C94.2,C94.3,C94.4,C96.0,C96.1,C96.2,C96.3,C96.4,C96.5,C96.6
	40006	C92,C92.0,C92.1,C92.2,C92.3,C92.4,C92.5,C92.6,C92.7,C92.8, C92.9,C93.0,C93.1,C93.2,C93.3,C93.7,C93.9,C94,C94.0,C94.1, C94.2,C94.3,C94.4,C96.0,C96.1,C96.2,C96.3,C96.4,C96.5,C96.6
	41203	205.0,202.3,202.5,202.6,205.0,205.1,205.2,205.3,205.8,205.9,20 6.0,206.1,206.2,206.8,206.9,207.0,207.1,207.2,238.4,238.71,238. 72,238.73,238.74,238.75,238.76
	41205	205.0,202.3,202.5,202.6,205.0,205.1,205.2,205.3,205.8,205.9,20 6.0,206.1,206.2,206.8,206.9,207.0,207.1,207.2,238.4,238.71,238. 72,238.73,238.74,238.75,238.76
Type 2 Diabetes	20002	1223
	41202	E11,E11.0,E11.1,E11.2,E11.3,E11.4,E11.5,E11.6,E11.7,E11.8,E 11.9
	41204	E11,E11.0,E11.1,E11.2,E11.3,E11.4,E11.5,E11.6,E11.7,E11.8,E 11.9
	40001	E11,E11.0,E11.1,E11.2,E11.3,E11.4,E11.5,E11.6,E11.7,E11.8,E 11.9
	40002	E11,E11.0,E11.1,E11.2,E11.3,E11.4,E11.5,E11.6,E11.7,E11.8,E 11.9

Supplemental Table 4: Panel of Antibodies used for the flow cytometry.

REAGENT or RESOURCE	SOURCE	IDENTIFIER
**Antibodies for Flow cytometry**		
**For mature myeloid staining**		
APC anti-mouse CD45.1 Antibody	BioLegend	Catalog No. 110714; Clone: A20; RRID: AB_313503
FITC anti-mouse CD45.2 Antibody	BioLegend	Catalog No. 109806; Clone: 104; RRID: AB_313443
PE anti-mouse/human CD11b Antibody	BioLegend	Catalog No. 101208; Clone: M1/70; RRID: AB_312791
APC/Cyanine7 anti-mouse Ly-6G/Ly-6C (Gr-1) Antibody	BioLegend	Catalog No. 108424; Clone: RB6-8C5; RRID: AB_2137485
PE/Cyanine7 anti-mouse CD3 Antibody	BioLegend	Catalog No. 100220; Clone: 17A2; RRID: AB_1732057
PerCP/Cyanine5.5 anti-mouse/human CD45R/B220 Antibody	BioLegend	Catalog No. 103236; Clone: RA3-6B2; RRID: AB_893354
**For Hematopoietic stem cells staining**		
PE anti-mouse Lineage Cocktail with Isotype Ctrl	BioLegend	Catalog No. 133303; Clones: 145-2C11; RB6-8C5; RA3-6B2; Ter-119; M1/70; RRID: AB_1595553
Brilliant Violet 785™ anti-mouse CD117 (c-kit) Antibody	BioLegend	Catalog No. 135138; Clone: ACK2; RRID: AB_2734197
PE/Cyanine7 anti-mouse Ly-6A/E (Sca-1) Antibody	BioLegend	Catalog No. 108114; Clone: D7; RRID: AB_493596
APC/Cyanine7 anti-mouse CD48 Antibody	BioLegend	Catalog No. 103432; Clone: HM48-1; RRID: AB_2561463
PerCP/Cyanine5.5 anti-mouse CD150 (SLAM) Antibody	BioLegend	Catalog No. 115922; Clone: TC15-12F12.2; RRID: AB_2303663
**For progenitor cells (CMP/GMP/MEP/CLP) staining**		
PE anti-mouse Lineage Cocktail with Isotype Ctrl	BioLegend	Catalog No. 133303; Clones: 145-2C11; RB6-8C5; RA3-6B2; Ter-119; M1/70; RRID: AB_1595553
Brilliant Violet 785™ anti-mouse CD117 (c-kit) Antibody	BioLegend	Catalog No. 135138; Clone: ACK2; RRID: AB_2734197
PE/Cyanine7 anti-mouse Ly-6A/E (Sca-1) Antibody	BioLegend	Catalog No. 108114; Clone: D7; RRID: AB_493596
APC/Cyanine7 anti-mouse CD16/32 Antibody	BioLegend	Catalog No. 101328; Clone: 93; RRID: AB_2104158
Brilliant Violet 421™ anti-mouse CD34 Antibody	BioLegend	Catalog No. 152208; Clone: SA376A4; RRID: AB_2650766
APC anti-mouse CD127 (IL-7Rα) Antibody	BioLegend	Catalog No. 121122; Clone: SB/199; RRID: AB_1595471
**For Myeloid blast staining**		
APC/Cyanine7 anti-mouse CD117 (c-kit) Antibody	BioLegend	Cat. No. 135136; Clone: ACK2; RRID: AB_2632809
PE anti-mouse/human CD11b Antibody	BioLegend	Catalog No. 101208; Clone: M1/70; RRID: AB_312791
**For Immunophenotyping**		

PE anti-mouse CD3 Antibody	BioLegend	Catalog No. 100206; Clone: 17A2; RRID: AB_312663
Brilliant Violet 421™ anti-mouse CD4 Antibody	BioLegend	Catalog No. 100438; Clone: GK1.5; RRID: AB_11203718
Brilliant Violet 605™ anti-mouse CD8a Antibody	BioLegend	Catalog No. 100744; Clone: 53-6.7; RRID: AB_2562609
PE/Cyanine7 anti-mouse CD279 (PD-1) Antibody	BioLegend	Catalog No. 135216; Clone: 29F.1A12; RRID: AB_10689635
APC-H7 Mouse Anti-Mouse CD366 (TIM-3)	BD Biosciences	Catalog No. 567165; Clone: 5D12/TIM-3; RRID: AB_2916482
APC anti-mouse IL-17A Antibody	BioLegend	Catalog No. 506916; Clone: TC11-18H10.1; RRID: AB_536018
